# Testing enabling techniques for olefin metathesis reactions of lipophilic substrates in water as a diluent

**DOI:** 10.1016/j.isci.2022.104131

**Published:** 2022-03-21

**Authors:** Agata Tyszka-Gumkowska, Vishal B. Purohit, Tomasz Nienałtowski, Michał Dąbrowski, Anna Kajetanowicz, Karol Grela

**Affiliations:** 1 Biological and Chemical Research Centre, Faculty of Chemistry, University of Warsaw, Żwirki i Wigury 101, 02-089 Warsaw, Poland; 2 Polpharma SA Pharmaceutical Works, Pelplińska 19, 83-200 Starogard Gdański, Poland

**Keywords:** Chemistry, Catalysis, Organic chemistry, Green chemistry

## Abstract

Olefin metathesis reactions of diverse polyfunctional substrates were conducted in water emulsions using two hydrophobic ruthenium catalysts in the presence of air. Instead of using surfactants to increase the efficiency of the metathesis reaction in water, ultrasound and microwave techniques were tested on a small-scale reaction, whereas conventional heating and mechanical stirring were effective enough to provide high conversion and selectivity on a larger scale. The developed conditions extend known protocols for the aqueous metathesis methodology, utilizing relatively low catalyst loadings and allowing for simple product isolation and purification. The established synthetic protocol was successfully adopted in the large-scale synthesis of a pharmaceutically related product – sildenafil (Viagra) derivative.

## Highlights

• Sustainable approach for metathesis reaction in water emulsion system on air.
• Utilization of enabling techniques for boosting metathesis under aqueous conditions.
• RCM of medically important sildenafil derivative.

## Graphical abstract

**Figure:**
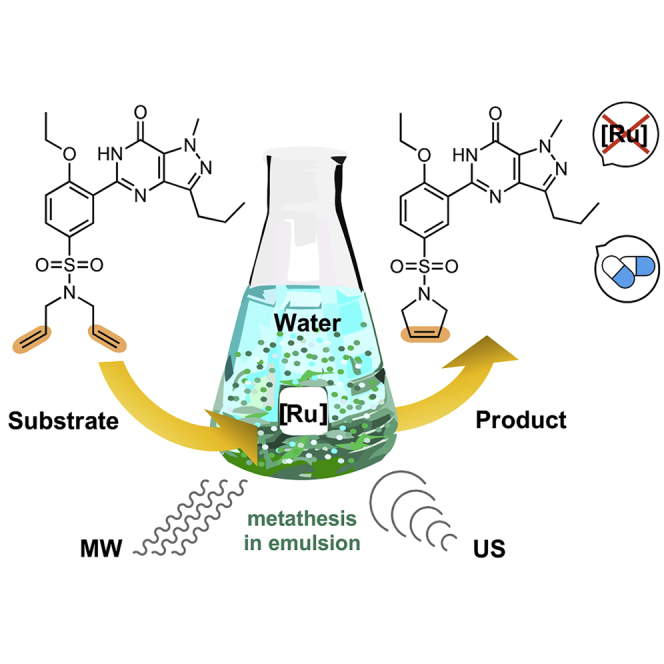

## Introduction

**Figure 1:**
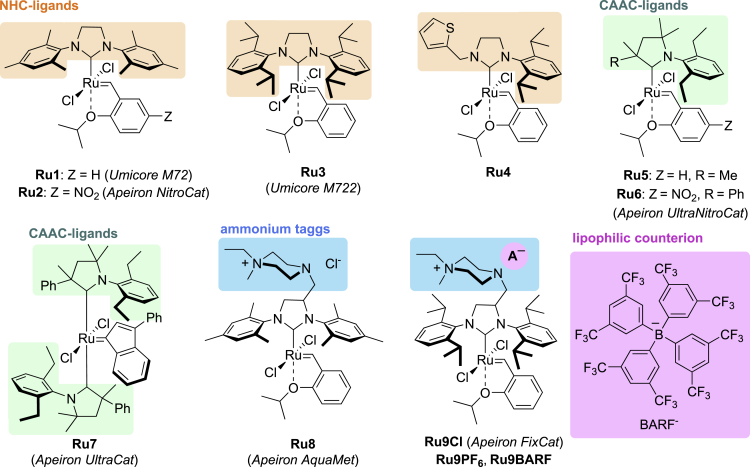
Popular water-insoluble catalysts Ru1–Ru7 and those designed for olefin metathesis in water Ru8–Ru9

Among the plethora methods for the construction of carbon-carbon double bonds, olefin metathesis has become one of the most convenient tool in chemists’ hands (Grela, 2014; Ogba et al., 2018). This is undoubtedly associated with the development of new ruthenium catalysts with increased resistance to air and moisture. Similarly to other organometallic transformations, metathesis reactions are preferably carried out in carefully dried and degassed solvents from a petrochemical sources (benzene, toluene, and chlorinated solvents). However, owing to environmental concerns, making the olefin metathesis suitable for Green Chemistry guidelines remains a great interest in the scientific community (Clavier et al., 2007). Therefore, several methods were already developed to facilitate this transformation in the green solvents, including aqueous media (Burtscher and Grela, 2009; Zaman et al., 2009), to minimize the amount of waste (Kniese and Meier, 2010), and heavy metal residues in products (Szczepaniak et al., 2015; Vougioukalakis, 2012; Wheeler et al., 2016). In particular, olefin metathesis in water (or in its mixtures with polar solvents) represents an important solution for biocompatible synthesis, as well as reactions of highly polar substrates, which suffer from low reactivity in the traditional organic media (Lipshutz and Ghorai, 2014; Sabatino and Ward, 2019). Unfortunately, serious limitations for using aqueous media in metathesis reactions are associated with poor solubility of the commercially available ruthenium catalysts in water. To address this issue, several tailor-made water-soluble catalysts have been introduced, obtained by decorating classical ruthenium catalysts with ionic tags (Skowerski et al., 2012; Wang et al., 2015), and with other polar groups, such as PEGs (Hong and Grubbs, 2006). Recently, some of such catalysts have been commercialized (e.g. Ru8, Ru9Cl Figure 1) (Olszewski et al., 2020).

On the opposite scale of this problem are the water-insoluble (hydrophobic) starting materials, for which water may also be a potentially interesting medium during metathesis reactions. A lot of efforts were devoted to find more environmentally friendly conditions for the transformation of these ubiquitous hydrophobic substrates. In particular, it was found that carrying out reactions “on water”, could be even more advantageous owing to the hydrophobic effect, which accelerates the reaction rate (Davis and Sinou, 2002). Nonetheless, reports on the use of common water insoluble metathesis catalysts under such heterogeneous aqueous conditions are still limited, probably owing to the problem of forming stable emulsions or micelles, facilitating the contact of catalyst with substrates. In a truly innovative approach, extensively explored by Lipshutz and co-workers, various amphiphilic additives (surfactants) allowed for performing the olefin metathesis reaction effectively “in water” (Lipshutz et al., 2008; Lipshutz and Ghorai, 2010). In this methodology, the addition of surfactants leads to the formation of micelles that can accommodate water-insoluble substrates along with a ruthenium metathesis catalyst. A similar strategy was applied in metathesis reactions with so-called *catsurfs* (catalyst + surfactant), specialized ruthenium-based catalysts that can act simultaneously as initiators and surfactants, thanks to their unique structure (Gawin et al., 2010). Although such catalysts are insoluble in water, after the addition of a lipophilic substrate, they promote the formation of a stable emulsion upon mechanical stirring. Nevertheless, relatively high loading of these tailor-made catalysts is usually required. Instead of using surfactants and *catsurfs*, olefin metathesis in emulsion can be obtained by ultrasonication of a lipophilic substrate and a commercially available hydrophobic catalyst floating on water (Gułajski et al., 2008, 2019). Under such conditions, the catalyst and substrates are encapsulated in small droplets formed in the reaction media, making the metathesis reaction proceed smoothly above the aqueous layer. These conditions seem to offer a convenient tool to conduct olefin metathesis with water used as reaction medium.

Surprisingly, with the exception of just a few reports (Gułajski et al., 2008, 2019; Sacco et al., 2015), ultrasounds are not frequently used in the context of olefin metathesis. This is probably connected with a number of limitations that remain unsolved, such as a relatively high loading of the ruthenium catalyst used in the reported cases and a limited substrate scope. Therefore, further development of olefin metathesis in aqueous media under environmentally mild, user-friendly, and economically viable conditions seems necessary.

Keeping in mind that the largest amount of materials (by mass or volume) used during almost any chemical reaction are solvents, reducing their amount is the key step in lowering the level of waste and upgrading EcoScale parameters of the process (Sheldon, 2007; Van Aken et al., 2006). For this reason, the solvent-free processes or those utilizing the minimal number of solvents (reactions at high concentration) are especially attractive in the industrial production. On the other hand, using none or only a small amount of solvent may cause a number of practical problems such as the risk of overheating, lack of reaction control, and increased risk of side product formation. In addition, in chemical transformations featuring solid starting materials or products, the difficulties in stirring the dense/pasty reaction mixture and problems in removing the product from the reactor shall be considered. In the particular case of olefin metathesis, faster decomposition of ruthenium catalyst under higher concentration can be expected sometimes, which might promote undesirable isomerization of substrates or products (Hong et al., 2005; Schmidt, 2004).

In this respect, it seems reasonable to us to use water as a diluent to suspend the reactants, ensuring convenient stirring and heat transfer. Moreover, in the context of waste management, water represents the major green solvent, since it is cheap, easily available, and non-toxic. However, the process of water purification is still expensive owing to relatively high heat capacity, which makes distillation processes energy consuming (Häckl and Kunz, 2018; Zhou et al., 2019). Therefore, the amount of water must be kept minimal whenever possible.

Herein we present our attempt for the olefin metathesis reactions under aqueous conditions, stressing the practical aspects of this processes, such as operational simplicity and efficient mass and heat transfer. These issues are crucial in performing reactions on a larger scale when one needs to consider not only the chemical but also the economical parameters of the transformation. To probe usefulness of the developed conditions on the manufacturing of fine chemicals and products of pharmaceutical interest, we decided to study a number of challenging lipophilic liquid and solid polyfunctional substrates in olefin metathesis using water as a reaction medium.

## Results and disscusion

### Optimization study of olefin metathesis reaction in the aqueous emulsion system

The development of ruthenium catalysts with increased stability and activity is one of the main trends in the advanced metathesis methodology. Peculiarly successful enhancement in activity has been achieved by introducing Hoveyda–Grubbs second-generation ruthenium complexes containing alkoxy styrene unit and N-heterocyclic carbenes (NHCs) as ligands, e.g. **Ru1**–**Ru4** (Garber et al., 2000; Scholl et al., 1999). High functional group tolerance allows for its wide-spread applications in the synthesis of natural products, pharmaceuticals and target materials under mild conditions (Nicolaou et al., 2005). Recently, catalysts containing cyclic (alkyl)(amino)carbenes (CAACs) e.g. **Ru5**–**Ru7** (Figure 1) also had significant impact in further extension of the metathesis methodology, especially in the industrial production of fine chemicals (Gawin et al., 2017; Kajetanowicz et al., 2020; Lavallo et al., 2005; Marx et al., 2015). Nevertheless, in the area of aqueous metathesis, the ability to tolerate water and air by the ruthenium species is still one of the major limitations (Blanco et al., 2021; Guidone et al., 2015; Ton and Fogg, 2019).

**Scheme 1:**
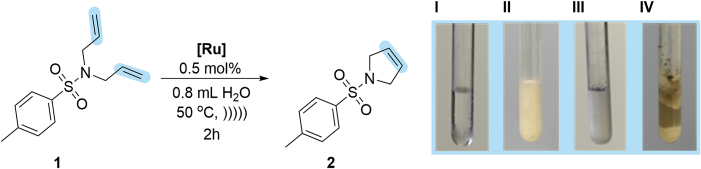
Model metathesis reaction in neat water, recorded at different stages of the reaction. (I): liquid substrate 1 under the surface of water; (II): emulsion formed after 5 min of sonication; (III): addition of a catalyst; (IV): reaction completed after 2 h of sonication and product 2 precipitation – ultrasounds

To select the most robust and active system in the envisioned emulsion protocol, we tested a series of selected ruthenium catalysts **Ru1**–**Ru9** in the ring closing metathesis (RCM) reaction of the standard model substrate, namely *N*,*N*-diallyltosylamide (**1**, DATA) floating under the water surface (Scheme 1). To ensure the best contact of the reactants, we used ultrasound that helped to create very small droplets of the organic phase and better stabilize the emulsion than mechanical stirring.

The catalytic activity of **Ru1**–**Ru9** was tested in *non-degassed distilled water, under air in a standard laboratory ultrasound bath*. More details concerning optimization studies are available in the supporting information (see Table S1), whereas the key results are summarized in Table 1.

**Table 1:** Screening of ruthenium catalysts Ru1–Ru9 in the RCM reaction of DATA in neat water in emulsion system; see also Table S1^a^

Entry	[Ru] catalyst (0.5 mol %)	Yield (%)^b^
1	**Ru1**	41
2	**Ru2**	67
3	**Ru3**	95
4	**Ru4**	28
5	**Ru5**	39
6	**Ru6**	77
7	**Ru7**	99 (99)^c^
8	**Ru8**	3
9	**Ru9Cl**	32
10	**Ru9PF**_**6**_	85
11	**Ru9BARF**	99 (99)^c^

a Reactions were carried out using 0.4 mmol of substrate in 0.8 mL of water at 50°C using acoustic sonication for emulsion formation.

b Calculated based on NMR experiments.

c 0.1 mol % of catalyst used.

Using commercially available catalysts **Ru1** and **Ru2**, we observed rather moderate yields in the benchmark reaction under ultrasound-supported emulsification conditions (Table 1, entries 1 and 2). Respectively, catalyst **Ru3** bearing a larger substituent in the NHC ligand showed much higher efficiency and gave the desired product in a 95% yield (Table 1, entry 3). This result is supported by higher stability and productivity generally observed for catalysts possessing a bulkier SIPr (1,3-bis-(2,6-triisopropylphenyl)-2-imidazolidinylidene) ligand in the structure (Clavier et al., 2009). Subsequently, we investigated the activity of catalyst **Ru4** with an unsymmetrically substituted NHC ligand (uNHC) (Monsigny et al., 2021a) and, unfortunately, we noted low substrate conversion (Table 1, entry 4). In the next attempt, CAAC derivatives **Ru5**, **Ru6**, and **Ru7** exhibiting relatively high protic solvent tolerance were tested (Blanco et al., 2021; Nagyházi et al., 2020). Owing to the superior activity of these ruthenium complexes connected with their slower initiation of metathetic cycle (Morvan et al., 2021a), we expected high activity in the RCM reaction of DATA (**1**). When **Ru5** gave rather low conversion of starting material, catalyst **Ru6** with electron withdrawing –NO_2_ group in alkoxybenzylidene ligand performed the RCM reaction of **1** in a 77% yield (Table 1, entries 5 and 6). Even better results were noted for catalyst **Ru7**, which gave the corresponding RCM product **2** in a quantitative yield (Table 1, entry 7). As mentioned above, an important factor determining the utility of a given ruthenium catalyst for metathesis in water emulsion systems is the stability of the resulting propagating catalytic species (Blanco et al., 2021). For this reason, the next logical step was to check the catalytic capability of catalysts **Ru8** and **Ru9** tailored for application in aqueous metathesis (the latter was additionally altered for the metathesis reaction in aqueous conditions by tuning its counter ion, *vide infra*). Surprisingly, for the fully water-soluble complex **Ru8**, we observed a very low conversion of model substrate **1** (Table 1, entry 8). We assumed that Cl^−^ counterion of **Ru8** is not lipophilic enough to provide good solubility of the catalyst in droplets of substrate **1**; therefore catalyst remains in an aqueous layer, which results in significant decrease in its activity. The second reason stands behind the lower performance of **Ru8** is unsuitable steric hindrance of the SIMes-type NHC ligand. In contrast, the polar but water-insoluble catalyst **Ru9Cl** bearing a bulkier SIPr-like NHC ligand led to a better result (Table 1, entry 9). This encouraged us to further tune the lipophilicity of this catalyst. To do so, we exchanged the chloride counter ion for more lipophilic PF_6_^−^ and BARF^−^ ones (for the procedure, see Star Method section), and subjected the obtained catalysts **Ru9PF**_**6**_ and **Ru9BARF** to the model metathesis reaction, observing increased productivity (Table 1, entries 10 and 11). Especially, the BARF complex (**Ru9BARF**) showed very high activity in the RCM reaction carried out in the emulsion system, giving the desired product in a 99% yield. Taking advantage of these results, for the most efficient complexes **Ru7** and **Ru9BARF**, we lowered the catalyst loading in the same benchmark reaction and found that both catalysts gave excellent results even at loadings as low as 0.1 mol % in neat water (Table 1, entries 7 and 11). It should be noted that the RCM reaction of DATA (**1**) at such low loadings was not reported in aqueous media so far. Nonetheless, experiments performed by Cazin et al. in boiling toluene and ppms of ruthenium catalyst (in open reaction vessels) are worth mentioning (Guidone et al., 2015).

We postulated that the high performance of the aforementioned catalysts in the emulsion system is connected either with the presence of two (lipophilic) CAAC ligands or the SIPr-like NHC ligand together with BARF counter ion. Such structural properties provide a strong lipophilic character to these complexes. These features aid in the diffusion of the catalyst into emulsion droplets, which not only enhances the catalysts interaction with a lipophilic substrate but also prevents rapid decomposition of the catalyst by water. To the best of our knowledge, these examples represent an exceptionally efficient application of relatively low loading of ruthenium-based catalysts under aqueous metathesis conditions without the use of external additives or surfactants. Moreover, using this protocol, it was possible to avoid unwanted isomerization of the double bond in the product (caused by the decomposition of the ruthenium catalyst into Ru-hydride complexes and other species) (Bailey et al., 2017), which was previously observed in reactions carried out under classic conditions (Liu et al., 2014; Qiao et al., 2012), especially in polar solvents (Bantreil et al., 2012; Skowerski et al., 2014).

### Scope and limitation study of aqueous emulsion olefin metathesis using ultrasounds

Encouraged by these results, we evaluated the most robust catalysts **Ru7** and **Ru9BARF** in a variety of olefin metathesis reactions in neat water using acoustic emulsification, and the result of these studies is summarized in Table 2.

**Table 2:** Ring-closing metathesis reactions of selected liquid and solid substrates catalyzed by Ru7 and Ru9BARF in neat water under acoustic sonication conditions^a^

Entry	Substrate	Product	Catalyst loading (mol %)	Time (h)	Yield (%) and E:Z ratio^b^
1			**Ru7** 0.5	2	97
			**Ru9BARF** 0.5	2	97
2			**Ru7** 1	3	90 99^c^
			****Ru9BARF**** 1	3	41 95^c^
3			**Ru9BARF** 1	5	0^c^
4			**Ru7** 1	5	97
			**Ru9BARF** 1	5	99
5			**Ru7** 1	5	99
			**Ru9BARF** 1	5	63
6			**Ru7** 1	5	99
			**Ru9BARF** 1	5	99
7			**Ru9BARF** 1	5	82
8			**Ru7** 1	5	86
9			**Ru7** 2.5	5	77
10			**Ru9BARF** 1	3	99^c^
11			**Ru9BARF** 2	8	17 98^c^

a Reactions were carried out using 0.4 mmol of substrate in 0.8 mL of water at 50°C using acoustic sonication for emulsion formation, reactants are marked as (S) = solid or (L) = liquid.

b Calculated based on NMR experiments.

c Reaction with 0.2 mL of AcOEt as a co-solvent.

The established conditions were first evaluated in a series of RCM reactions with various dienes. We started our studies from classic substrate diethyl diallylmalonate (**3**) and observed that reaction proceed smoothly in neat water using only 0.5 mol % of catalyst **Ru7** or **Ru9BARF** (Table 2, entry 1). Similar result was noted for more demanding compound **5**, when 1 mol % of **Ru7** was enough to obtain a 90% yield of the desired product **6** (Table 2, entry 2). Interestingly, the addition of a small amount of ethyl acetate as a co-solvent allowed us to achieved quantitative course of this transformation. An increased of the yield was especially visible in the case of **Ru9BARF** (from 41% in neat water to 95% with co-solvent). Whereas liquid and lipophilic substrates gave us gratifying results in RCM reactions leading to a five-membered ring, polar one was not reactive under examined conditions (Table 2, entry 3). With these types of water-soluble compounds, the reaction could take place only in the region between an organic and aqueous phase because formed emulsion droplets contain only catalyst molecules. In this scenario, the probability of reaction between polar and highly solvated substrate and non-soluble in the water catalyst is dramatically lower. This fact also convinces us that emulsification of the reaction mixture is beneficial for rather lipophilic substrates, and the methodology is not suitable for highly polar substrates.

Next, we decided to focus on substrates known as more reluctant in RCM reactions. For this purpose, we choose dienes leading to products with trisubstituted C-C double bonds **9** and **11**, and were pleased to obtain the cyclisation products **10** and **12** in very high yields in the presence of only 1 mol % **Ru7** or **Ru9BARF** catalyst (Table 1, entries 4 and 5). Also, the RCM reaction of **13** giving a six-membered ring product **14** proceeds in almost a quantitative yield with low loading of tested catalysts **Ru7** and **Ru9BARF** (Table 2, entry 6).

On the basis of these results, we proceeded to extend the scope of the method’s applicability to include products with seven-membered rings. In the reaction of compound **15**, we noted an 82% yield in the formation of 1-tosyl-2,3,6,7-tetrahydro-1*H*-azepine (**16**) with only 1 mol % of **Ru9BARF** (Table 2, entry 7). In further research, we focused also on pharmaceutically related substrates to emphasize the practical aspect of the emulsion system under study. Therefore, we tested the RCM reaction of allyl(1-methylpent-4-enyl)carbamic acidbenzyl ester (**17**), which led to precursor of Relacatib—drug with high potency to inhibit cathepsin K that prevents bone resorption (Yamashita et al., 2006). In this case, a very good yield (86%) can be achieved after a 5 h reaction with 1 mol % of **Ru9BARF** (Table 2, entry 8). Another important example is the synthesis of **20**, precursor of a Halidor possessing antispasmodic, vasodilator, and platelet aggregation inhibitor properties (Martin et al., 1974). In this case, reactions also proceed smoothly in neat water using a low loading of catalyst **Ru7** (Table 2, entry 9).

These gratifying results allowed us to attempt RCM reactions of more demanding starting materials related to medicinal chemistry that possess a number of functional groups with Brønsted basic sites that can potentially bind to ruthenium complex and obstruct catalytic cycle. To this aim, diallylated derivatives of ketamine **21** and sildenafil (Viagra) **23** were subjected to the emulsion methodology using **Ru9BARF** catalyst (Table 2, entries 10 and 11). It also should be noted that both substrates are solid materials, forming suspensions in water, which could impede their access to the catalyst (also forming an insoluble suspension in water). Therefore, to increase the reagent contact during these RCM reactions, the addition of a small amount of ethyl acetate as a co-solvent was necessary. Despite the presence of an organic co-solvent, the crude products **22** and **24** precipitated from the reaction mixtures and were easily isolated in the high yield by simple filtration and drying.

The positive results of the above experiments encouraged us to investigate the even more difficult cross-metathesis (CM) reactions. The self-cross-metathesis (self-CM) of allylbenzene (**25**) worked similarly well, as clean formation of the desired product **26** in a high yield was observed using again a relatively low catalyst **Ru7** loading of 0.5 mol % (Table 3, entry 1). Similarly, self-CM of functionalized allylbenzenes (phenylpropenoids), eugenol (**27**), and eugenol acetate (**29**), resulted in clean formation of the expected products, however only in moderate yield (54–56%) despite addition of the co-solvent (Table 3, entries 2 and 3). One of the challenges in the above-mentioned reactions is the known predisposition of the phenylpropenoid substrates to undergo isomerization of the C-C double bond during olefin metathesis (Małecki et al., 2019). It is noteworthy that by-products derived from isomerization processes were not detected under the emulsion conditions, in contrast to reactions carried out in classic organic solvents when only the use of a specialized catalyst prevented by-products formation and gave satisfactory results. As with the RCM reactions, now we also observed that water-soluble substrates such as allyl alcohol (**31**) were not reactive in the emulsion system when the hydrophobic catalyst was used. In contrast, the CM of lipophilic substrates, such as the reaction between 6-chloro-hexene (**33**) and a sterically demanding partner (an excess of 4-methyl-1-pentene, **34**), proceeded in water in a quantitative yield using 1 mol % of catalysts **Ru7** or **Ru9BARF** (Table 3, entry 5). It has also been found that allylbenzene (**25**) under acoustic emulsification conditions undergoes a highly efficient CM reaction with *cis*-1,4-diacetoxy-2-butene (**36**), without isomerization of **37** to the corresponding styrene derivative (Małecki et al., 2019). Seeing these encouraging results, we switched to more challenging CM reactions, including an electron-deficient partners. Under aqueous conditions, this type of CM reaction sometimes leads to lower conversion and selectivity and usually requires higher loading of the olefin metathesis catalyst (Connon and Blechert, 2003). The results obtained for CM reaction of 1-octene (**38**) with α,β-unsaturated aldehyde **39** showed high efficiency under discussed conditions (Table 3, entry 7).

**Table 3:** Cross-metathesis reactions of selected liquid and solid substrates catalyzed by Ru7 and Ru9BARF in neat water under acoustic sonication conditions^a^

Entry	Substrate	Product	Catalyst loading (mol %)	Time (h)	Yield (%) and E:Z ratio^b^
1			**Ru7** 0.5	2	87 8:2
2			**Ru7** 1	2	56^c^ 8.5:1.5
3			**Ru7** 2	2	54^c^ 6:4
4			**Ru9BARF** 1	5	0
5			**Ru7** 1	5	99^c^ 8:2
			**Ru9BARF** 1	5	99^c^ 8.5:1.5
6			**Ru9BARF** 1	5	90^c^ 7.5:2.5
7			**Ru9BARF** 1	5	99

a Reactions were carried out using 0.4 mmol of substrate in 0.8 mL of water at 50°C using acoustic sonication for emulsion formation, reactants are marked as (S) = solid or (L) = liquid.

b Calculated based on NMR experiments.

c Reaction with 0.2 mL of AcOEt as co-solvent.

In a separate study, we have attempted to CM reactions of 9-decen-1-ol (**41**) with various olefins: **42**, **39**, and **34**. The tested alcohol has an amphiphilic character, which makes it somewhat difficult to carry out the reaction in water. Although the obtained results are in general acceptable, to ensure good selectivity and to avoid the parasitic self-CM process (homodimerization) of alcohol **41**, a catalyst loading higher than 2.5 mol % with simultaneous addition of a small volume of the co-solvent was required (Table 4, column A). To overcome the observed limitation, we decided to further modify the reaction protocol by using the well-established microwave technique (Kirschning et al., 2006; Tagliapietra et al., 2020).

**Table 4:** RCM and CM reaction of 9-decen-1-ol (41) with different partners catalyzed by Ru9BARF in the presence of ultrasounds ( )))); column A)^a^ and microwaves (MW; column B)^b^

Entry	Substrates	Product	Catalyst loading (mol %)	Yield (%) E:Z ratio^c^	
				A^a^ ))))	**B**^b^ MW
1			**Ru9BARF** 2.5	57 9:1	87 9:1
2			**Ru9BARF** 1	85	93
3			**Ru9BARF** 1	66 2.5:7.5	89 3:7
4			**Ru9BARF** 1	63	75
5			**Ru9BARF** 1	99	99

a Reaction carried out using ultrasound sonication conditions (H_2_O:AcOEt, 0.8:0.2 mL, 50°C, 5 h) reactants are marked as (S) = solid or (L) = liquid.

b Reaction carried out in microwave reactor (0.8 mL H_2_O, 20 W, 50°C, 30 min).

c Calculated based on NMR experiments.

### Scope and limitation study of metathesis reactions using microwave irradiation in water

A well-known alternative strategy to perform metathesis reactions of demanding substrates is the use of microwave irradiation (Bantreil et al., 2012; Coquerel and Rodriguez, 2008; Ullah and Arshad, 2017), especially in expensive and not environmentally benign fluorinated aromatic solvents (Samojłowicz et al., 2011b). In many cases, the use of this thermal activation technique has a general beneficial effect in terms of shorter reaction times, slower catalyst degradation, and less by-products formation (Kappe, 2019; Kappe et al., 2013). Utilization of water as a solvent during experiments in microwave reactor could be particularly profitable, owing to the excellent heat and energy transfer offer by this polar (and green) solvent (Dallinger and Kappe, 2007). Surprisingly, examples describing metathesis reactions in the aqueous media under microwave irradiation conditions are scarce and thus worth of further investigations (Castagnolo et al., 2009; Gułajski et al., 2019). In view of these considerations, we examined the potential synergetic effect of aqueous conditions with microwave irradiation in previously problematic CM reaction of 9-decen-1-ol (**41**) with olefins **42**, **39**, and **34**. Remarkably, microwave assistance allows for a significant increase of yields and decrease of time in tested reactions (Table 3, column B). Furthermore, a CM carried out under microwave irradiation does not require the addition of ethyl acetate as co-solvent, which positively affects environmental concerns.

In particular, the CM of alcohol **41** with methyl acrylate (**42**) using microwave activation allows obtaining the desired product in an 87% yield after only 30 min. In the same reaction performed under standard emulsion conditions, significant amounts of unreacted substrate and homodimerization side-product were observed, with a final yield of **43** that is equal to 57% after 5 h (Table 4, entry 1). Similar observations were made for CM reactions with electron-deficient crotyl aldehyde (**39**) and sterically demanding 4-methyl-1-pentane (**34**). Gratifyingly, for these transformations, even a lower catalyst **Ru9BARF** loading (1 mol %) was sufficient to achieve a high yield in the microwave reactor (Table 4, entries 2 and 3). The formation of trisubstituted C-C double bonds was accomplished under both ultrasound and microwave conditions (Table 4, entries 4–5). Although these reactions gave very good yields under standard emulsion conditions, diene **11** demonstrated a several percent increase in the yield under microwave conditions.

**Scheme 2:**
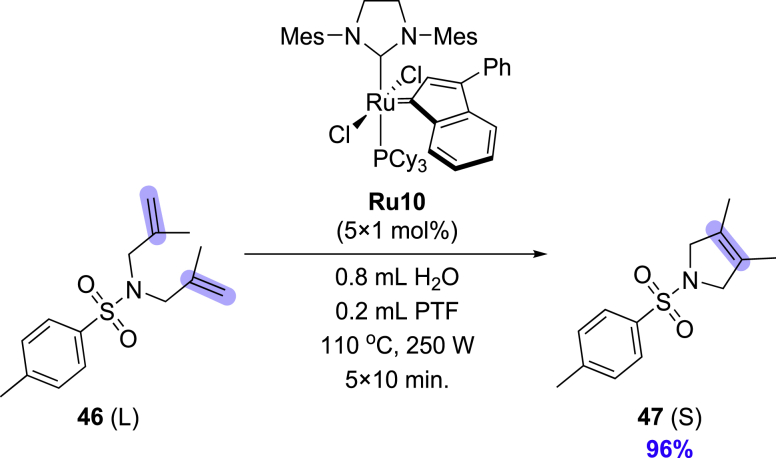
Preparation of compound 47 in a microwave reactor using 0.2 mmol of substrate in 1 mL of water:PFT mixture (8:2, v/v); reactants are marked as (S) = solid or (L) = liquid

Tetrasubstituted C-C double bonds are considered one of the least reactive substrates in olefin metathesis reaction (Heinrich et al., 2020; Lecourt et al., 2018; Mukherjee et al., 2018). Whereas molybdenum alkylidene complexes (Schrock catalysts) are very effective in this case, ruthenium carbenes are less reactive and require specially designed sterically reduced NHC ligands (Berlin et al., 2007; Kuhn et al., 2010; Stewart et al., 2007) or forcing conditions (Peeck and Plenio, 2010; Sashuk et al., 2010; Urbina-Blanco et al., 2013). According to our knowledge, such reactions were never attempted in water. Therefore, we decided to investigate the performance of various Ru-catalysts in RCM, leading to the formation of a tetrasubstituted olefin **47**. Unfortunately, quick reconnaissance shown that a specialized ruthenium catalyst with a small NHC ligand (Planer et al., 2020) decomposes very quickly under reaction conditions in water. Similarly, **Ru9BARF** with a sterically augmented SIPr-like ligand gave no conversions in the case of the formation of a tetrasubstituted C-C double bond. Therefore, we decided to use the SIMes-based catalyst Umicore Grubbs M202 (**Ru10**), and as a co-solvent, we selected perfluorotoluene (PFT) because it was previously found to give very good results in challenging formation of crowded C-C double bonds by olefin metathesis (Rost et al., 2008; Samojłowicz et al., 2011a, 2011b; Szadkowska et al., 2011). Using microwave irradiation to provide high temperature in the aqueous emulsion system, we conducted a model reaction of diene **46** (Scheme 2) observing 70% of conversion. Interestingly, under precisely the same conditions, but with PFT replaced by ethyl acetate, only 30% conversion was reached, which shows that indeed PFT accelerates this transformation (for details, see Supporting information, Scheme S1). Finally, we were able to run this reaction to completion (96% of **47**), however under more forcing conditions (5 × 1 mol % of **Ru10**, H_2_O/PFT 8:2 v/v, 110°C, 250 W, 50 min). This result shows that water can be used as a diluent also in the formation of tetrasubstituted bonds, further allowing to reduce the volume of PFT typically used (under previously reported conditions, PFT is used as the only solvent in a larger volume (Wang et al., 2021), but here it acts only as a co-solvent added in a small amount of water).

Undoubtedly, these all examples underline the advantages of microwave irradiation in the water-based heterogeneous metathesis system and represent an interesting example of a challenging metathesis reaction using this enabling technique.

### Scale-up and application prospects of metathesis in water (use of mechanical stirring and testing practicality of product separation)

**Scheme 3:**
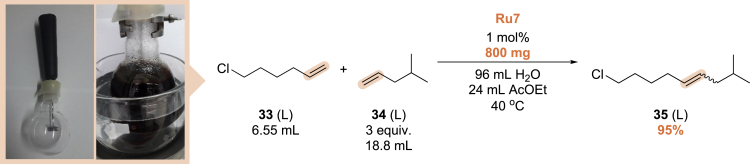
Large-scale preparation of compound 35 in the emulsion system using a milk frother: reaction setup (left picture) and emulsion formed after 5 min of shaking (right picture); reactants are marked as (S) = solid or (L) = liquid

During the previous screening studies, products were usually isolated by extraction with ethyl acetate and purified by column chromatography. Obviously, such a separation technique is limited only to academic research and small-scale experiments. Interestingly, in many previously discussed reactions in water emulsions, we observed the formation of oily product droplets on the surface of water, directly after ending the sonication, or after removing the sample from the microwave reactor. This reveals the possibility of avoiding extraction in reactions carried out with water as a diluent *on a larger scale*. To demonstrate the practical application of the developed method, we attempted the previously tested (cf. Table 3, entry 5) CM reaction of 6-chloro-hex-1-ene (**33**) with 4-methyl-pente-1-ene (**34**) in a 150 mL flask using a simple milk frother to form an emulsion (Scheme 3). After the completion of the reaction, the water-insoluble product (**35**) was isolated by decantation from the post-reaction mixture in 95% purity. Presented examples indeed shows that simple and convenient phase separation works well when water is used as the medium for the metathesis reaction.

**Scheme 4:**
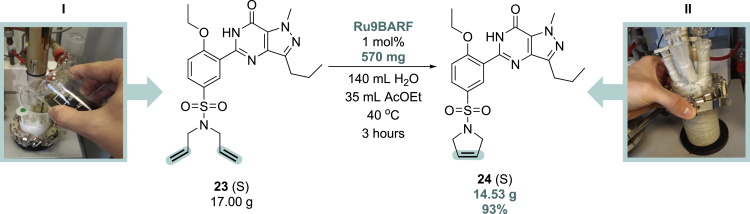
Large-scale preparation of compound 24 in the emulsion system, recorded at different stages of the reaction. (I): charging reactor with substrate, catalyst, and solvents; (II): completion of the reaction and precipitation of the product; reactants are marked as (S) = solid

When metathesis transformations of pharmaceutical interest substrates are attempted on a larger scale, several practical (technical) aspects need to be addressed, such as optimizing product separation and purification, including rigorous removal of ruthenium traces. In cooperation with a pharmaceutical company, we decided to check if water can be used as a diluent for the olefin metathesis reaction in the pharmaceutical context. To do so, we focused on a preparative synthesis of sildenafil derivative **24** in a larger amount, using techniques and hardware typical for pharmaceutical R + D scale (Scheme 4). In the first step, the Mettler Toledo Easymax workstation reactor equipped with a mechanical stirrer was charged with 17.0 g of a solid sildenafil derivative **23** and water (140 mL). The materials were weighted, and the reactor was charged open to air. This heterogeneous mixture was stirred for several minutes to form a stable suspension of the (water insoluble) substrate, then a solution of (water insoluble) catalyst **Ru9BARF** in ethyl acetate (35 mL) was added. Next, the reaction mixture was stirred at 40°C for 3 h, until full conversion of the substrate was indicated by TLC analysis. Cooling down of the post-reaction mixture led to the formation of a precipitate that after filtration and drying in the binder vacuum dryer gave 14.5 g of the final product as a beige solid in a 94% yield.

Since ruthenium catalysts have found wide application in the synthesis of active pharmaceutical ingredients (APIs), certain restrictions regarding the heavy metal content in the final product are enforced by national and international regulators. Therefore, the legal limit of transition metal contamination shall typically be less than 10 ppm in the final API (European Medicines Agency, 2008). In addition to ruthenium toxicity, the presence of still-active or even decomposed catalyst causes the risk of isomerization, polymerization, or even degradation of the metathesis product during work-up (Clavier et al., 2007). Whereas several diverse methods for ruthenium scavenging were tested in the context of pharmaceutical production (Vougioukalakis, 2012; Wheeler et al., 2016), according to our knowledge, no attempts to purify a pharmaceutically relevant product obtained in aqueous metathesis have been reported so far. To do so, using the sample of the sildenafil derivative obtained in the previous experiment (Scheme 3), we decided to study this issue. First, using ICP-MS technique, we determined ruthenium contamination in the crude product 10 precipitated from the aqueous emulsion. Then the crude product was divided into four portions and each of them was purified using different protocols; the results are summarized in Table 5.

**Table 5:** Attempts for purification of compound 24 after large-scale RCM reaction in water emulsion

Entry	Purification method	Trace Ru content [ppm]
1	Theoretical amount of ruthenium used in reaction (1 mol %)	10,000^a^
2	Crude product as precipitated from the aqueous reaction mixture	1,032
3	Single crystallization from ethyl acetate and ethanol	8
4	Treatment with SnatchCat metal scavenger and crystallization	5
5	Treatment with charcoal and crystallization	4

For detailed procedures, see the STAR Methods section.

a Calculated based on catalyst loading.

The crude product 24 was found to contain already a reduced level of ruthenium (1,032 ppm) as compared to the initial amount of ruthenium used for reaction (Table 5, entries 1 and 2). Fortunately, simple recrystallization of the crude product from a mixture of ethyl acetate and ethanol resulted in significant decrease of product contamination (Table 5, entry 3). An alternative purification protocol, consisting of the use of a ruthenium scavenger developed in our laboratories (Szczepaniak et al., 2019; Toh et al., 2021) (recently commercialized under the trade name SnatchCat), led to 5 ppm of ruthenium residue (Table 5, entry 4). Interestingly, similar results were obtained after treatment of crude compound **24** (containing 1,032 of ruthenium) with active charcoal (Table 5, entry 5).

The following observations can be noted: (a) interestingly, spontaneous precipitation of product out from the aqueous reaction mixture does not occlude a large amount of catalysts, despite the low solubility in water of the catalyst used; (b) a simple crystallization reduced the ruthenium contamination below the pharmacopoeia accepted levels; (c) a commercially available metal scavenger and active charcoal were similarly effective in further removal of ruthenium level in the crude product. Therefore, it seems that water can be a convenient solvent (diluent) even for solid, polyfunctional substrates exhibiting a high level of molecular complexity, and the ruthenium species formed after catalyst decomposition do not severely contaminate the crude product, which can be purified with relative ease.

It shall be stressed that in both large-scale attempts, we observed almost identical yields compared with the previous small-scale experiments (cf. Table 2, entry 11 and Table 3, entry 5). It should be also noted that despite the high masses of the catalysts used in our studies (**Ru7**_MW_ = 1,062 g/mol, **Ru9BARF**_MW_ = 1,716 g/mol), owing to relatively low loadings, they constitute approximately ∼4–6 mass-% of the final products. We think that these simple experiments demonstrate the scale-up prospects of the presented methodology, easy product separation and purification, and highlight the utility of water as the reaction medium (or: *diluent*) in olefin metathesis.

## Conclusions

We presented a simple synthetic protocol for the preparation of various functionalized products by RCM and CM reactions in a water emulsion system under mild conditions using sonication, microwaves assistance, or mechanical stirring, on air and without using an additional surfactant. In comparison with previously developed protocols for aqueous metathesis reactions, the introduced method applies a relatively low loading of two hydrophobic metathesis catalysts. The studied conditions demonstrate a broad application profile. Moreover, the reactions can be easily scaled up without drop of the yield. In particular, experiments on a larger scale (17 g) have shown the convenience of the elaborated methodology in the synthesis of a pharmaceutically related derivative of sildenafil (Viagra) in an excellent yield and with a low level of ruthenium contamination after simple purification. Therefore, we believe that water as a diluent can find broad applications in olefin metathesis reactions, characterized by a low amount of waste produced, a low loading of the catalyst, and a high selectivity.

### Limitations of the study

Despite advantages of the developed methodology, the scope of substrates is limited mainly to the lipophilic non-ionic compounds as for the water-soluble substrates, metathesis reactions were not effective. Moreover, in such cases, the isolation of final product might require either distillation or crystallization.

## STAR★Methods

### Key resources table

**Table:** 

REAGENT or RESOURCE	SOURCE	IDENTIFIER
**Chemicals, peptides, and recombinant proteins**		

Ru1 (1,3-Bis-(2,4,6-trimethylphenyl)-2-imidazolidinylidene)dichloro(o-isopropoxyphenylmethylene)ruthenium	Sigma Aldrich	Cat#569755 CAS: 301224-40-8
Ru2 1,3-dimesitylimidazolidin-2-ylidene)dichloro(2-isopropoxy-5-nitrobenzylidene)ruthenium(II)	Apeiron Synthesis	Cat#AS2032 CAS: 502964-52-5
Ru3 Dichloro[1,3-bis(2,6-isopropylphenyl)-2-imidazolidinylidene](2-isopropoxyphenylmethylene)ruthenium(II)	Sigma Aldrich	cat code: 729345 CAS: 635679-24-2
Ru4	Synthetized in our lab	https://doi.org/10.1002/chem.201803460
Ru5 1-(2,6-diethylphenyl)-3,3,5,5-tetramethylpyrrolidin-2-ylidene)dichloro(2-isopropoxy-5-benzylidene)ruthenium(II)	Synthetized in our lab	https://doi.org/10.1002/anie.201609009
Ru6 (1-(2,6-diethylphenyl)-3,5,5-trimethyl-3-phenylpyrrolidin-2-ylidene)dichloro(2-isopropoxy-5-nitrobenzylidene)ruthenium(II)	Apeiron Synthesis	Cat#AS2091 CAS: 2106819-64-9
Ru7 bis(1-(2,6-diethylphenyl)-3,5,5-trimethyl-3-phenylpyrrolidin-2-ylidene)dichloro(3-phenyl-1H-inden-1-ylidene)ruthenium(II)	Apeiron Synthesis	Cat#AS2086 CAS: 2055540-61-7
Ru8 (4-((4-ethyl-4-methylpiperazin-1-ium-1-yl)methyl)-1,3-dimesitylimidazolidin-2-ylidene)dichloro(2-isopropoxybenzylidene)ruthenium(II) chloride	Apeiron Synthesis	Cat#AS2038 CAS: 1414707-08-6
Ru9Cl (1,3-bis(2,6-diisopropylphenyl)-4-((4-ethyl-4-methylpiperazin-1-ium-1-yl)methyl)imidazolidin-2-ylidene)dichloro(2-isopropoxybenzylidene)ruthenium(II) chloride	Apeiron Synthesis	Cat#AS2061 CAS: 1799947-97-9
Ru9PF_6_ (1,3-bis(2,6-diisopropylphenyl)-4-((4-ethyl-4-methylpiperazin-1-ium-1-yl)methyl)imidazolidin-2-ylidene)dichloro(2-isopropoxybenzylidene)ruthenium(II) hexafluorophosphate	Apeiron Synthesis	Cat#AS2083 CAS: 2249721-31-9
Ru9BARF (1,3-bis(2,6-diisopropylphenyl)-4-((4-ethyl-4-methylpiperazin-1-ium-1-yl)methyl)imidazolidin-2-ylidene)dichloro(2-isopropoxybenzylidene)ruthenium(II) tetrakis[3,5-bis(trifluoromethyl)phenyl]borate	Synthetized in our lab	this work
SnatchCat	Apeiron Synthesis	Cat#AS1033 CAS: 51641-96-4
NaBARF	Apollo Scientific	Cat#PC1999 CAS: 79060-88-1
1 *N*,*N*-diallyltosylamide	Synthetized in our lab	https://doi.org/10.1039/B911999J
3 diallylmalonic acid diethyl ester	Synthetized in our lab	https://doi.org/10.1021/ol0712632
5 2-phenyl-2-(2-propenyl)-4-pentenenitrile	Synthetized in our lab	https://doi.org/10.1039/C3CC48874H
7 diallyldimethylammonium chloride	Sigma Aldrich	Cat#32598 CAS: 7398-69-8
9 *N*-allyl-4-methyl-*N*-(2-methylallyl)benzenesulfonamide	Synthetized in our lab	https://doi.org/10.1021/ja037394p
11 ethyl-(2-methyl-allyl)-malonic acid diethyl ester	Synthetized in our lab	https://doi.org/10.1021/ol402339e
13 2-allyl-2-but-3-enyl-malonic acid diethyl ester	Synthetized in our lab	https://doi.org/10.1039/c4ob02480j
15 *N*,*N*-di(but-3-enyl)-4-methylbenzenesulfonamide	Synthetized in our lab	https://doi.org/10.1002/chem.200700256
17 allyl(1-methylpent-4-enyl)carbamic acid benzyl ester	Synthetized in our lab	https://doi.org/10.1021/jm050915u
19 5-benzylnona-1,8-dien-5-ol	Synthetized in our lab	https://doi.org/10.1021/acs.joc.7b02468
21 *N*,*N*-diallyl-2-(1H-indol-3-yl)-2-oxoacetamide	Synthetized in our lab	https://doi.org/10.1002/chem.201604934
23 4-ethoxy-3-(1-methyl-7-oxo-3-propyl-6,7-dihydro-1H-pyrazolo[4,3-d]pyrimidin-5-yl)- *N*,*N*-di(prop-2-en-1-yl)benzenesulfonamide	Pharmaceutical Works Polpharma SA	provided by pharmaceutical company
25 allylbenzene	abcr	Cat#AB131212 CAS: 300-57-2
27 4-allylguaiacol	Sigma Aldrich	Cat#E51791 CAS: 97-53-0
29 methyl 4-propenyl-2-methoxyphenylfuran-2-acetate	Sigma Aldrich	Cat#04733 CAS: 93-28-7
31 allyl alcohol	Sigma Aldrich	Cat#240532 CAS: 107-18-6
33 6-chlorohexene	Alfa Aesar	Cat#H53396.14 CAS: 928-89-2
34 4-methyl-1-pentene	Sigma Aldrich	Cat#M67400 CAS: 691-37-2
36 cis-1,4-bis(acetyloxy)but-2-ene	Tokyo Chemical Industry	Cat#D1358 CAS: 25260-60-0
38 oct-1-ene	Alfa Aesar	Cat#A11146.AP CAS: 111-66-0
39 trans-crotonaldehyde	Sigma Aldrich	Cat#262668 CAS: 123-73-9
41 9-decen-1-ol	Tokyo Chemical Industry	Cat#D1892 CAS: 13019-22-2
42 acrylic acid methyl ester	Acros Organics	Cat#126195000 CAS: 292638-85-8
46 *N*-tosyldimethallylamine	Synthetized in our lab	https://doi.org/10.1002/adsc.200505447

**Other**		

Thin layer chromatography using TLC silica gel plates with fluorescent indicator (l = 254 nm)	Merck Millipore	https://merckmillipore.com
silica gel (60, particle size 0.043 – 0.063 nm).	Merck Millipore	https://merckmillipore.com
Agilent Mercury 400 MHz spectrometer	Agilent	www.agilent.com
inductively coupled plasma mass spectrometer PC-MS, NexION 300D	PerkinElmer	www.perkinelmer.com
milk frother	Tchibo	www.tchibo.pl
ultrasonic bath Elmasonic S 120 (220-240 V)	Elmasonic	www.elma-ultrasonic.com
Microwave reactor CEM Discover SP	CEM	www.cem.com

### Resource availability

#### Lead contact

Further information and requests for resources and reagents should be directed to and will be fulfilled by the lead contact, Anna Kajetanowicz (a.kajetanowicz@uw.edu.pl).

#### Materials availability

All other data supporting the finding of this study are available within the article and the Supplemental information or from the lead contact upon reasonable request.

#### Data and code availability

• All data reported in this paper will be shared by the lead contact upon request.
• This paper does not report original code.
• Any additional information required to reanalyse the data reported in this paper is available from the lead contact upon request

### Method details

#### General remarks

All materials were purchased from commercial suppliers and used as received. The bottles with ruthenium catalysts were stored under argon atmosphere, but no special precautions were taken to avoid air or moisture exposure in the moment of extracting catalysts from the bottles.

Analytical thin-layer chromatography (TLC) was performed using silica gel 60 F254 precoated plates (0.25 mm thickness, Merck) with a fluorescent indicator. Visualization of TLC plates was performed by KMnO_4_ aqueous solution and anisaldehyde/H_2_SO_4_ stain. The flash column chromatography was performed using Merck silica gel 60 (230–400 mesh) with *n*-hexane/ethyl acetate eluent system, unless otherwise stated.

NMR spectra were recorded on Agilent 400-MR DD2 400 MHz spectrometer. NMR chemical shifts are reported in ppm with solvent residual peak as a reference. Deuterated solvents was purchased from Eurisotop, stored over molecular sieves and used without further purification. The following abbreviations are used in reporting NMR data: s (singlet), d (doublet), t (triplet), q (quartet), quint (quintet), sex (sextet), sep (septet), m (multiplet), br (broad). ^1^H NMR signals are given followed by multiplicity, coupling constants J in Hertz, and integration in parentheses. The obtained data was processed with the software MestReNova. High Resolution Mass Spectra (HRMS) were provided by the analytical laboratory at the Institute of Biochemistry and Biophysics, PAS. Inductively Coupled Plasma Mass Spectrometry (ICP-MS) measurements of ruthenium content was determined using NexION 300D apparatus (Perkin Elmer, USA).

#### Synthesis of **Ru9BARF**

**Figure:**
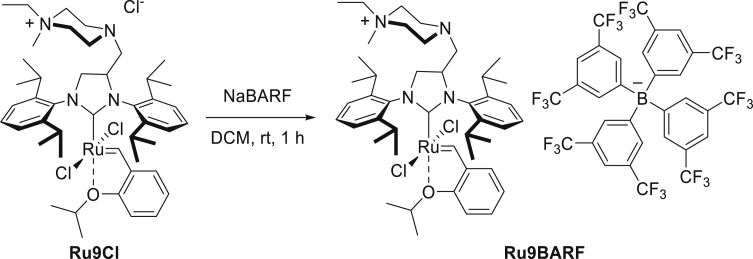

Using Schlenk technique, NaBARF (250 mg, 0.282 mmol) was mixed with 70 mL of anhydrous DCM under argon atmosphere (NaBARF was not fully dissolve). After 15 min of stirring FixCat **Ru9Cl** (1.0 equiv., 250 mg, 0.282 mmol) was added and reaction mixture was stirring for 1 h at room temperature. The mixture was concentrated on rotary evaporator and then placed on alumina long pad (Brockmann activity IV) and washed with DCM. Highly movable green band was collected. Solution of product was evaporated and dried under high vacuum yielding product **Ru9BARF** as a green powder (413 mg, 0.241 mmol, 86%). ^1^H NMR (400 MHz, CD_2_Cl_2_) δ 16.12 (s, 1H), 7.73 (s, 8H), 7.65 (t, *J* = 7.6 Hz, 1H), 7.57 (s, 4H), 7.56–7.49 (m, 2H), 7.45 (d, *J* = 7.7 Hz, 2H), 7.42–7.33 (m, 2H), 6.93–6.78 (m, 3H), 5.01–4.84 (m, 1H), 4.48–4.27 (m, 2H), 4.22–4.02 (m, 1H), 3.84–3.67 (m, 1H), 3.63–3.53 (m, 1H), 3.53–3.36 (m, 1H), 3.32–3.25 (m, 6H), 2.93 (s, 3H), 2.84–2.76 (m, 3H), 2.69–2.59 (m, 1H), 1.57–1.53 (m, 8H), 1.47–1.43 (m, 2H), 1.36–1.22 (m, 22H), 1.13–1.07 (m, 2H), 0.89–0.83 (m, 2H). ^13^C NMR (101 MHz, CD_2_Cl_2_) δ 289.5, 217.6, 162.1 (q,^1^*J*_*C-B*_ = 49.8 Hz), 152.7, 149.5, 144.0, 137.0, 135.2, 130.5, 130.4, 130.1, 129.2 (q, ^2^*J*_*C-F*_ = 31.7 Hz), 126.0, 125.7, 125.0 (q, ^1^*J*_*C-F*_ = 272.5 Hz), 124.5, 122.9, 122.7, 117.9, 113.4, 75.7, 63.4, 60.8, 60.1, 53.8, 47.6, 46.8, 29.3, 28.2, 25.7, 24.3, 23.2, 22.0, 7.7. ^19^F NMR (376 MHz, CD_2_Cl_2_) δ −62.8. ^11^B NMR (128 MHz, CD_2_Cl_2_) δ −6.6. HRMS ESI: positive (*m*/*z*) calc. for C_45_H_67_Cl_2_N_4_ORu^+^ [M]^+^ 851.3730, found 851.3727; negative (*m*/*z*) calc. for C_32_H_12_BF_24_^⁻^ [M]^-^ 863.0643, found 863.0662.

#### General procedure for metathesis in emulsion with ultrasound’s assistance

A reaction vial charged with substrates (0.4 mmol), durene as internal standard (0.4 mmol, 54.2 mg, 1 equiv.) and distilled water (0.8 mL) was sonicated for 5 min in 50°C. To the resulting suspension, the ruthenium catalyst soluble in 0.2 mL of AcOEt or in solid form was added and sonicated for specified time (between 2 and 5 h). After that time solution of SnatchCat in 4 mL of AcOEt (4.4 equiv. counting on amount of the catalyst) and 1 mL of brine was added. Phase was separated and extracted with AcOEt (2 × 4 mL). Organic phase was dried over NaSO_4_, then filtrated and volatiles were removed under reduced pressure to yield crude product which was analyzed using ^1^H NMR technique. Products **2**-**40** were obtained using above-described method.

#### Characterization of products **2**-**40**

##### *1-tosyl-2,5-dihydro-1H-pyrrole* (**2**)

**Figure:**
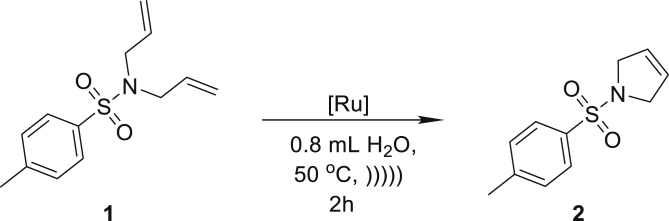

^1^H NMR (400 MHz, CDCl_3_) δ 7.76–7.69 (m, 2H), 7.35–7.28 (m, 2H), 5.65 (m, 2H), 4.12 (m, 4H), 2.43 (s, 3H). ^13^C NMR (101 MHz, CDCl_3_) δ 143.4, 134.2, 129.7, 127.4, 125.4, 54.8, 21.5; melting point: 120°C. Presented analytical data are consistent with literature (Szczepaniak et al., 2018).

##### *diethyl cyclopent-3-ene-1,1-dicarboxylate* (**4**)

**Figure:**
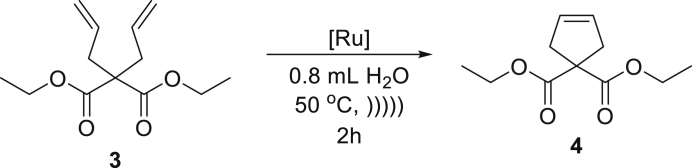

^1^H NMR (400 MHz, CDCl_3_) δ: 5.60 (m, 2H), 4.20 (q, *J* = 7.1 Hz, 4H), 3.01 (m, 4H), 1.25 (t, *J* = 7.1 Hz, 6H). ^13^C NMR (101 MHz, CDCl_3_) δ: 172.2, 127.8, 61.5, 58.8, 40.8, 14.0. Presented analytical data are consistent with literature (Do et al., 2015).

##### *1-phenylcyclopent-3-enecarbonitrile* (**6**)

**Figure:**
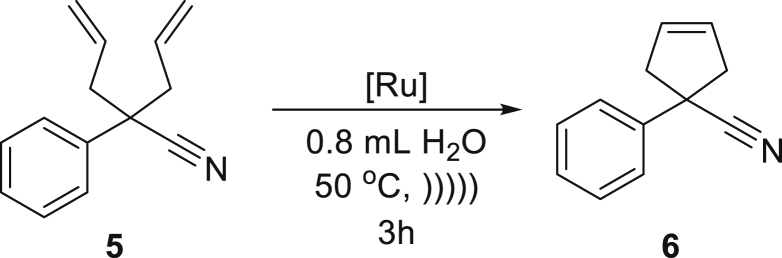

^1^H NMR (400 MHz, CDCl_3_) δ 7.50–7.45 (m, 2H), 7.43–7.35 (m, 2H), 7.35–7.28 (m, 1H), 5.83 (m, 2H), 3.37–3.24 (m, 2H), 3.03–2.88 (m, 2H). ^13^C NMR (101 MHz, CDCl_3_) δ 141.4, 128.9, 128.4, 127.7, 125.3, 124.8, 48.4, 44.9. Presented analytical data are consistent with literature (Szczepaniak et al., 2018).

##### *3-methyl-1-tosyl-2,5-dihydro-1H-pyrrole* (**10**)

**Figure:**
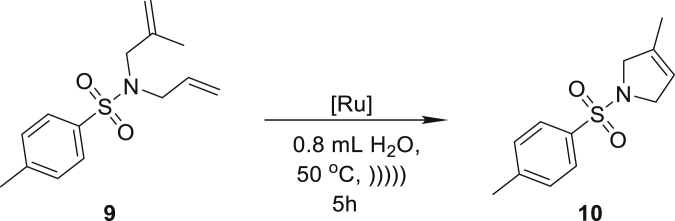

^1^H NMR (400 MHz, CDCl_3_) δ 7.73–7.68 (m, 2H), 7.34–7.28 (m, 2H), 5.26–5.21 (m, 1H), 4.09–4.03 (m, 2H), 3.99–3.92 (m, 2H), 2.41 (s, 3H), 1.66–1.60 (m, 3H). ^13^C NMR (101 MHz, CDCl_3_) δ 143.3, 135.0, 134.2, 129.7, 127.4, 119.0, 57.7, 55.1, 21.5, 14.0. Presented analytical data are consistent with literature (Szczepaniak et al., 2019).

##### *diethyl3-methylcyclopent-3-ene-1,1-dicarboxylate* (**12**)

**Figure:**
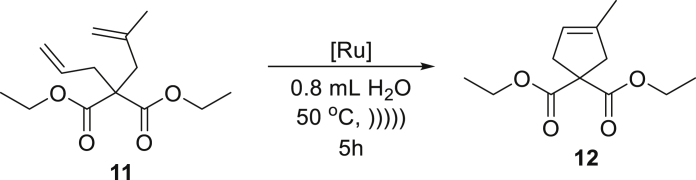

^1^H NMR (400 MHz, CDCl_3_) δ: 5.24–5.11 (m, 1H), 4.18 (q, *J* = 7.1 Hz, 4H), 3.05–2.92 (m, 2H), 2.92–2.84 (m, 2H), 1.77–1.62 (m, 3H), 1.23 (t, *J* = 7.1 Hz, 6H).^13^C NMR (101 MHz, CDCl_3_) δ: 172.4, 137.4, 121.3, 61.4, 59.4, 44.6, 40.8, 16.0, 14.0. Presented analytical data are consistent with literature (Monsigny et al., 2021a, 2021b).

##### *diethyl cyclohex-3-ene-1,1-dicarboxylate* (**14**)

**Figure:**
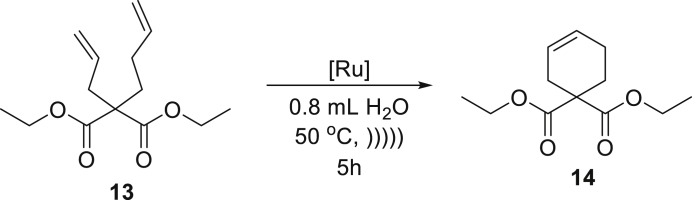

^1^H NMR (400 MHz, CDCl_3_) δ: 5.70–5.61 (s, 2H), 4.17 (qd, *J* = 7.1, 1.2 Hz, 4H), 2.60–2.49 (s, 2H), 2.18–2.00 (m, 4H), 1.23 (t, *J* = 7.1 Hz, 6H). ^13^C NMR (101 MHz, CDCl_3_) δ: 171.6, 126.0, 124.0, 61.2, 52.9, 30.4, 27.3, 22.3, 14.0. Presented analytical data are consistent with literature (Che et al., 2009; Monsigny et al., 2021a, 2021b).

##### *1-tosyl-2,3,6,7-tetrahydro-1H-azepine* (16)

**Figure:**
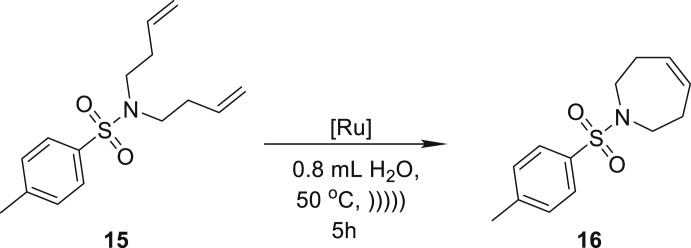

^1^H NMR (400 MHz, CDCl_3_) δ 7.70–7.63 (m, 2H), 7.32–7.27 (m, 2H), 5.77–5.72 (m, 2H), 3.29–3.23 (m, 4H), 2.42 (s, 3H), 2.34–2.28 (m, 4H). ^13^C NMR (101 MHz, CDCl_3_) δ 143.1, 136.2, 130.2, 129.7, 127.0, 48.3, 29.9, 21.5. Presented analytical data are consistent with literature (Michrowska et al., 2006).

##### *2-methyl-2,3,4,7-tetrahydro-azepine-1-carboxylic acid benzyl ester* (18)

**Figure:**
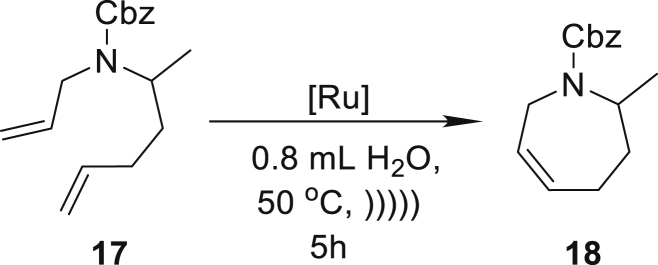

^1^H NMR (400 MHz, CDCl_3_) δ 7.38–7.30 (m, 5H), 5.75–5.60 (m, 2H), 5.17–5.11 (m, 2H), 4.46–4.05 (m, 2H), 3.66–3.54 (m, 1H), 2.27–2.07 (m, 2H), 1.94–1.76 (m, 2H), 1.14 (dd, *J* = 6.4, 3.6 Hz, 3H). ^13^C NMR (101 MHz, CDCl_3_) δ 156.2, 156.1, 137.2, 137.1, 131.7, 131.4, 128.4, 128.3, 127.8, 127.7, 127.6, 127.4, 66.9, 66.7, 52.5, 52.3, 39.4, 39.1, 34.0, 33.9, 27.1, 26.9, 19.6, 19.1.Presented analytical data are consistent with literature (Szczepaniak et al., 2019; Toh et al., 2021).

##### *1-benzylcyclohept-4-en-1-ol* (**20**)

**Figure:**
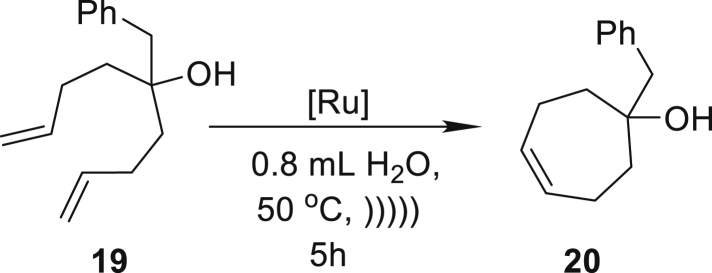

^1^H NMR (400 MHz, CDCl_3_) δ 7.36–7.24 (m, 5H), 5.77–5.73 (m, 2H), 2.81 (s, 2H), 2.37–2.24 (m, 2H), 2.09–1.94 (m, 2H), 1.70–1.63 (m, 4H). ^13^C NMR (101 MHz, CDCl_3_) δ 137.2, 131.6, 130.7, 128.1, 126.5, 74.9, 48.5, 38.5, 23.0. Presented analytical data are consistent with literature (Rodriguez et al., 2020; Toh et al., 2021).

##### *1-(2,5-dihydro-1H-pyrrol-1-yl)-2-(1H-indol-3-yl)ethane-1,2-dione* (**22**)

**Figure:**
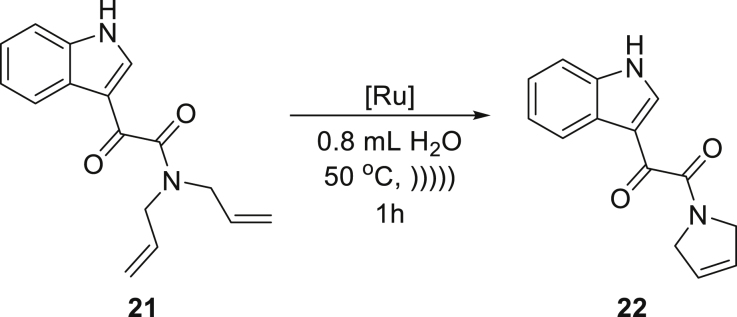

^1^H NMR (400 MHz, CDCl_3_) δ 9.04 (s, 1H), 8.41 (m, 1H), 8.27 (d, *J* = 3.2 Hz, 1H), 7.42 (m, 1H), 7.38–7.28 (m, 2H), 5.96–5.77 (m, 2H), 4.57–4.37 (m, 4H). ^13^C NMR (101 MHz, CDCl_3_) δ 136.2, 136.2, 125.9, 125.8, 124.7, 124.2, 123.2, 122.3, 111.6, 54.0, 53.4; melting point: 216°C. Presented analytical data are consistent with literature (Nienałtowski et al., 2020b).

##### *5-(5-((2,5-dihydro-1H-pyrrol-1-yl)sulfonyl)-2-ethoxyphenyl)-1-methyl-3-propyl-1,6-dihydro-7H pyrazolo[4,3-d]pyrimidin-7-one* (**24**)

**Figure:**
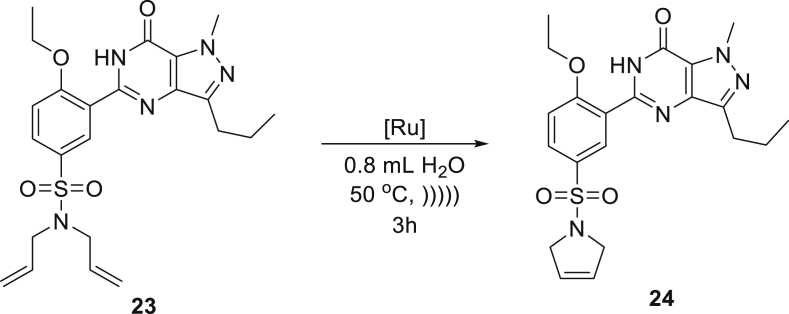

^1^H NMR (400 MHz, CDCl_3_) δ 10.82 (s, 1H), 8.89 (d, *J* = 2.4 Hz, 1H), 7.92 (dd, *J* = 8.8, 2.4 Hz, 1H), 7.15 (d, *J* = 8.8 Hz, 1H), 5.69 (s, 2H), 4.37 (q, *J* = 7.0 Hz, 2H), 4.27 (s, 3H), 4.19 (s, 4H), 2.93 (t, *J* = 7.4 Hz, 2H), 1.92–1.79 (m, 2H), 1.63 (t, *J* = 7.0 Hz, 3H), 1.03 (t, *J* = 7.4 Hz, 3H). ^13^C NMR (101 MHz, CDCl_3_) δ 159.1, 153.6, 146.9, 146.5, 138.3, 131.3, 131.0, 130.5, 125.5, 124.5, 121.1, 113.1, 66.0, 55.0, 38.2, 27.7, 22.3, 14.6, 14.0; melting point: 187°C. Presented analytical data are consistent with literature (Monsigny et al., 2021a, 2021b).

##### *1,4-diphenylbut-2-ene* (**26**) (mixture of *E* and *Z* isomers)

**Figure:**
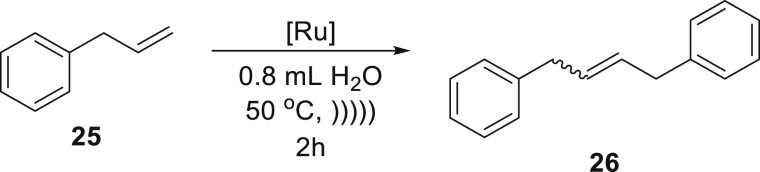

^1^H NMR (400 MHz, CDCl_3_) isomer *E* (80%) δ 7.34–7.28 (m, 4H), 7.24–7.15 (m, 6H), 5.68 (ddd, *J* = 5.3, 3.7, 1.6 Hz, 2H), 3.38 (dd, *J* = 3.7, 1.5 Hz, 4H); *Z* (20%) δ 7.34–7.28 (m, 4H), 7.24–7.15 (m, 6H), 5.72 (m, 2H) 3.53 (d, *J* = 5.6 Hz, 4H). ^13^C NMR (101 MHz, CDCl_3_) isomer *E:* δ 140.7, 130.4, 128.5, 128.4, 125.9, 38.9, isomer *Z:* 140.8, 129.0, 128.5, 128.46, 125.9, 33.5. Presented analytical data are consistent with literature (Keitz et al., 2011; Morvan et al., 2021b).

##### *4,4'-(but-2-ene-1,4-diyl)bis(2-methoxyphenol)* (**28**) (mixture of *E* and *Z* isomers)

**Figure:**
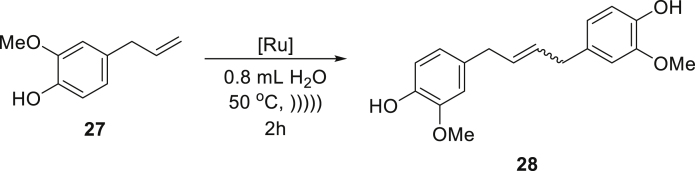

^1^H NMR (400 MHz, CDCl_3_) δ 6.84 (d, *J* = 4.0 Hz, 2H), 6.82 (d, *J* = 3.9 Hz, 2H), 6.71–6.66 (m, 2H), 5.72–5.67 (isomer *Z* (15%), m, 0.3H), 5.67–5.60 (isomer *E* (85%), m, 1.7H), 3.85 (isomer *E* (85%), s, 5.1H), 3.83 (isomer *Z* (15%), s, 0.9H), 3.32 (d, *J* = 6.7 Hz, 4H). Presented analytical data are consistent with literature (Alexander et al., 2016).

##### *but-2-ene-1,4-diylbis(2-methoxy-4,1-phenylene) diacetate* (**30**) (mixture of *E* and *Z* isomers)

**Figure:**
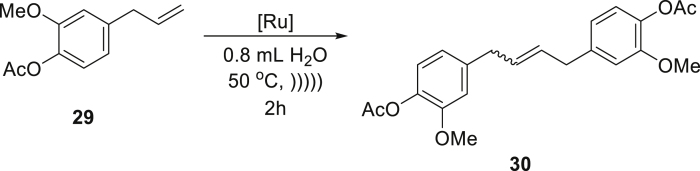

^1^H NMR (400 MHz, CDCl_3_) δ 6.92 (s, 2H), 6.79 (q, *J* = 2.1 Hz, 4H), 5.75 (isomer *Z* (40%), m, 0.8H), 5.69 (isomer *E* (60%), m, 1.2H), 3.80 (s, 3H), 3.41–3.31 (m, 4H), 2.32–2.28 (m, 6H). ^13^C NMR (101 MHz, CDCl_3_) δ 169.3, 151.0, 150.9, 139.7, 139.6, 137.9, 137.9, 130.5, 129.1, 122.6, 122.5, 120.5, 120.4, 112.7, 112.5, 55.8, 38.9, 33.3, 20.7. Presented analytical data are consistent with literature (Nienałtowski et al., 2020b).

##### *9-chloro-2-methylnon-4-ene* (**35**) (mixture of *E* and *Z* isomers)

**Figure:**
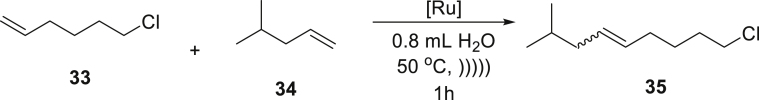

^1^H NMR (400 MHz, CDCl_3_) δ 5.52–5.28 (m, 2H), 3.54 (td, *J* = 6.7, 1.1 Hz, 2H), 2.09–1.98 (m, 2H), 1.94–1.83 (m, 2H), 1.83–1.71 (m, 2H), 1.63–1.45 (m, 3H), 0.89 (isomer *Z* (15%), d, *J* = 6.6 Hz, 0.9H), 0.87 (isomer *E* (85%), d, *J* = 6.6 Hz, 5.1H). ^13^C NMR (101 MHz, CDCl_3_) isomer *E* δ: 130.5, 130.2, 45.0, 42.1, 36.4, 32.0, 31.8, 28.5, 26.8, 22.3; isomer *Z* δ: 130.2, 129.8, 45.0, 41.9, 36.4, 32.0, 31.7, 28.4, 26.7, 22.3. Presented analytical data are consistent with literature (Nienałtowski et al., 2020a).

##### *4-Phenylbut-2-en-1-yl acetate* (**37**) (mixture of *E* and *Z* isomers)

**Figure:**
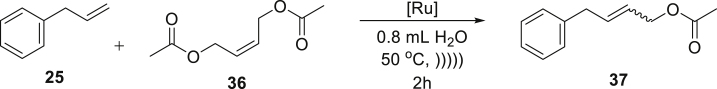

^1^H NMR (400 MHz, CDCl_3_) δ 7.31–7.23 (m, 2H), 7.22–7.12 (m, 3H), 5.99–5.89 (isomer *E* (75%), m, 0.75H), 5.89–5.80 (isomer *Z* (25%), m, 0.25H), 5.67–5.56 (m, 1H), 4.79–4.72 (isomer *Z* (25%), m, 0.5H), 4.61–4.51 (isomer *E* (75%), m, 1.5H), 3.49 (isomer *Z* (25%), d, *J* = 7.6 Hz, 0.5H), 3.42 (isomer *E* (75%), d, *J* = 6.8 Hz, 1.5H), 2.09 (isomer *Z* (25%), s, 0.75H), 2.09 (isomer *E* (75%), s, 2.25H). ^13^C NMR (101 MHz, CDCl_3_) isomer *E* δ 170.8, 139.5, 134.5, 128.6, 128.5, 126.2, 125.2, 64.9, 38.7, 21.0; isomer *Z* δ 170.9, 139.8, 133.4, 128.6, 128.4, 128.2, 126.2, 125.3, 124.2, 60.2, 33.8, 21.5. Presented analytical data are consistent with literatur (Karras et al., 2018).

##### *8-phenyl-oct-5-enal* (**40**)

**Figure:**
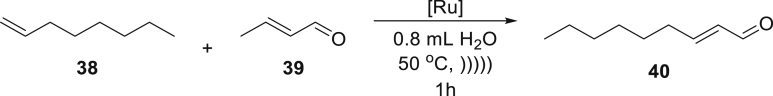

^1^H NMR (400 MHz, CDCl_3_) δ 9.51 (d, *J* = 7.9 Hz, 1H), 6.85 (dt, *J* = 15.6, 6.8 Hz, 1H), 6.12 (ddt, *J* = 15.6, 7.9, 1.5 Hz, 1H), 2.39–2.28 (m, 2H), 1.55–1.46 (m, 2H), 1.38–1.28 (m, 6H), 0.90 (t, *J* = 6.6 Hz, 3H). ^13^C NMR (101 MHz, CDCl_3_) δ 194.3, 159.2, 132.9, 32.7, 31.5, 28.8, 27.8, 22.5, 14.0. Presented analytical data are consistent with literature (Zhu et al., 2013).

#### General Procedure for metathesis in emulsion and microwaves assistance

A reaction vial charged with substrates (0.4 mmol), durene as internal standard (0.4 mmol, 54.2 mg, 1 equiv.), distilled water (0.8 mL), and ruthenium catalyst was placed into microwave reactor and irradiated with 20 W for 30 min at 50°C. After that time solution of SnatchCat in 4 mL of AcOEt (4.4 equiv. counting on amount of the catalyst) and 1 mL of brine was added. Phases were separated and extracted with AcOEt (2x4 mL). Organic phase was dried over NaSO_4_, then filtrated, and volatiles were removed under reduced pressure to yield crude product which was analyzed using ^1^H NMR technique. Products **43**-**46** were obtained using above-described method.

#### Characterization of products **43**-**46**

##### *11-hydroxy-undec-2-ensaeure-methylester* (**43**) (mixture of *E* and *Z* isomers)

**Figure:**
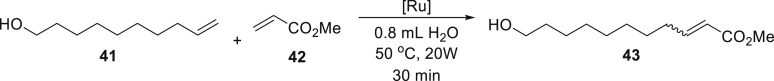

^1^H NMR (400 MHz, CDCl_3_) δ 6.96 (dt, *J* = 15.5, 7.0 Hz, 1H), 5.82 (isomer *E* (90%), dt, *J* = 15.6, 1.6 Hz, 0.9H), 5.77 (isomer *Z* (10%), dt, *J* = 11.5, 1.6 Hz, 0.1H), 3.73 (isomer *E* (90%), s, 2.7H), 3.71 (isomer *Z* (10%), s, 0.3H), 3.63 (t, *J* = 6.6 Hz, 2H), 2.18 (td, *J* = 8.0, 1.2 Hz, 2H), 1.54 (dd, *J* = 13.9, 6.9 Hz, 2H), 1.49–1.38 (m, 2H), 1.30 (m, 8H). ^13^C NMR (101 MHz, CDCl_3_) isomer *Z* + *E* δ 167.2, 149.8, 120.8, 63.0, 51.4, 32.7, 32.2, 29.3, 29.25, 29.0, 27.9, 25.7. Presented analytical data are consistent with literature (Diallo et al., 2010).

##### *11-hydroxyundec-2-enal* (**44**)

**Figure:**
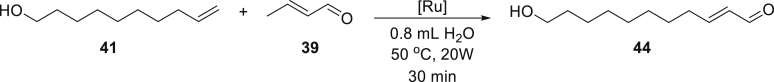

^1^H NMR (400 MHz, CDCl_3_) δ 9.50 (d, *J* = 7.9 Hz, 1H), 6.85 (dt, *J* = 15.6, 6.8 Hz, 1H), 6.11 (ddt, *J* = 15.6, 7.9, 1.5 Hz, 1H), 3.64 (t, *J* = 6.6 Hz, 2H), 2.39–2.24 (m, 2H), 1.60–1.44 (m, 4H), 1.40–1.28 (m, 8H). ^13^C NMR (101 MHz, CDCl_3_) δ 194.2, 159.0, 132.9, 63.0, 32.71, 32.70, 29.28, 29.25, 29.0, 27.8, 25.7. Presented analytical data are consistent with literature (Aubineau and Cossy, 2018).

##### *(9)-12-methyltridec-9-en-1-ol* (**45**) (mixture of *E* and *Z* isomers)

**Figure:**
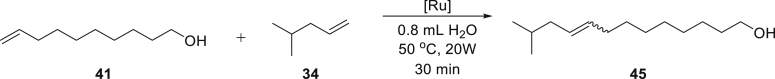

^1^H NMR (400 MHz, CDCl_3_) δ 5.45–5.26 (m, 2H), 3.63 (t, *J* = 6.6 Hz, 2H), 2.00 (m, 2H), 1.93–1.80 (m, 2H), 1.65–1.48 (m, 3H), 1.30 (m, 12H), 0.89 (isomer *Z* (25%), d, *J* = 6.8 Hz, 1.5H), 0.89 (isomer *E* (75%), d, *J* = 6.6 Hz, 4.5H). ^13^C NMR (101 MHz, CDCl_3_) *E*-isomer: δ 131.5, 129.0, 63.1, 42.0, 32.7, 29.6, 29.4, 29.4, 29.0, 28.5, 25.7, 22.3. *Z*-isomer: δ 130.5, 128.5, 63.1, 42.0, 36.4, 29.7, 29.6, 29.5, 29.3, 29.2, 27.3, 22.4. HRMS ESI (*m*/*z*) calc. for C_14_H_29_O ([M]^+^H)^+^ 213.2213, found 213.2211.

##### *3,4-dimethyl-1-[(4-methylphenyl)sulfonyl]-2,5-dihydro-1H-pyrrole* (47)

**Figure:**
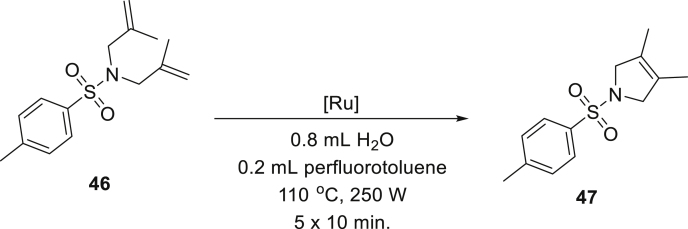

^1^H NMR (400 MHz, CDCl_3_) δ δ 7.75–7.68 (m, 2H), 7.35–7.28 (m, 2H), 3.97 (s, 4H), 2.43 (s, 3H), 1.54 (s, 6H) ^13^C NMR (101 MHz, CDCl_3_) δ 143.3, 134.2, 129.8, 127.6, 126.3, 59.039, 21.9, 11.5. Presented analytical data are consistent with literature (Wu et al., 2012).

#### Procedure for scale-up RCM reaction of sildenafil derivative **24**

**Figure:**
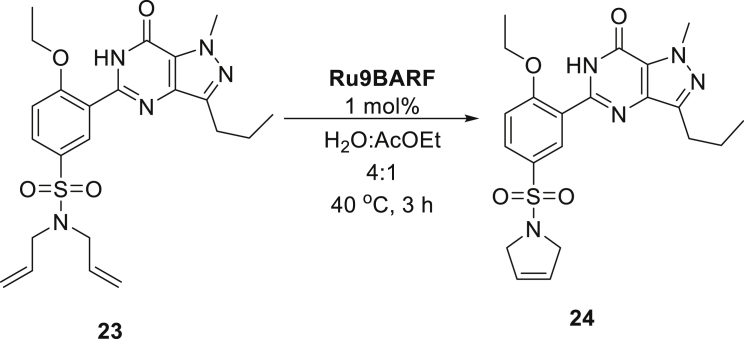

To a suspension of 4-ethoxy-3-(1-methyl-7-oxo-3-propyl-6,7-dihydro-1H-pyrazolo[4,3-d]pyrimidin-5-yl)-*N,N*-di(prop-2-en-1-yl)benzenesulfonamide (**23**) (17 g, 40 mmol) in distilled water (140 mL) solution of the catalyst **Ru9BARF** (1 mol%, 0.57 g) in ethyl acetate (35 mL) was added. The reaction mixture was vigorously stirred (600 RPM – anchor type stirrer) at 40°C for three hours, until TLC monitoring showed complete conversion. The precipitated product was filtered and dried in vacuum drier. 5-[5-(2,5-dihydro-1H-pyrrol-1-ylsulfonyl)-2-ethoxyphenyl]-1-methyl-3-propyl-1,6-dihydro-7*H*-pyrazolo[4,3-d]pyrimidin-7-one (**24**) was obtained as a beige solid (14.53 g, 93%) containing 1032 ppm of Ru. The product was divided into three fractions (1,2, and 3) to test different possibilities of removing traces of ruthenium:

1) Resulted product **24** (4.5 g) was dissolved in ethyl acetate:ethanol (8:2) mixture at reflux and cooled down to 0-5 °C. The precipitated product was filtered and dried in vacuum drier to obtain product as a beige solid (4.2 g, 93%) containing 8 ppm of Ru.
2) Resulted product **24** (4.5 g) was dissolved in ethyl acetate:ethanol (8:2) mixture at reflux, then SnatchCat (116 mg) was added and reaction mixture was stirred for 1 hour. Precipitated residues were filtered off and solution was cooled down to 0-5 °C. The precipitated product was filtered and dried in vacuum drier to obtain product as a cream solid (4.0 g, 89%) containing 5 ppm of Ru.
3) Resulted product **24** (4.5 g) was dissolved in ethyl acetate:ethanol (8:2) mixture at reflux and activated charcoal (1 gram) was added, then reaction mixture was stirred for one hour. Residues were filtered off and solution was cooled down to 0-5°C. The precipitated product was filtered and dried in vacuum drier to obtain product as a cream solid (4.1 g, 91%) containing 4 ppm of Ru.

#### Procedure for scale-up CM reaction of **33** and **34**

**Figure:**
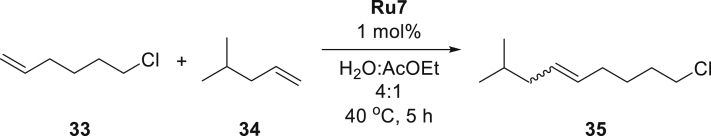

Suspension of 6-chlorohexene (**33**) (6.55 mL, 48 mmol, 1 equiv.) and 4-methyl-1-pentene (**34**) (48.8 mL, 144 mmol, 3 equiv.) in distilled water (96 mL) was vigorously stirred for 5 minutes by milk frother in 150 mL round bottom flask at 40°C. To formed emulsion a solution of the technical grade catalyst **Ru7** (60% purity, 1 mol%, 800 mg) in ethyl acetate (24 mL) was added. The reaction mixture was vigorously stirred at 40°C for five hours. After that time SnatchCat (464 mg) was added and reaction mixture was stirred for additional 1 h. Then organic phase was separated, dried over NaSO_4_, then filtrated, and volatiles were removed under reduced pressure to yield crude product 35 (8.9 mL, 95% yield).

## Supplemental information

Document S1. Figures S1–S5, Table S1, and Scheme S1
