# Supplementary material for: Testing enabling techniques for olefin metathesis reactions of lipophilic substrates in water as a diluent
Source: iScience. 2022 Mar 21;25(4):104131. doi: 10.1016/j.isci.2022.104131 (PMC9010768; doi:10.1016/j.isci.2022.104131)
Supplement: Document S1. Figures S1–S5, Table S1, and Scheme S1 [file mmc1.pdf]

iScience, Volume *25*

Supplemental information

Testing enabling techniques for olefin metathesis reactions of lipophilic substrates in water as a diluent

Agata Tyszka-Gumkowska, Vishal B. Purohit, Tomasz Nienałtowski, Michał Dąbrowski, Anna Kajetanowicz, and Karol Grela

Optimization studies

Table S1. Effect of various ruthenium catalysts on RCM reaction of *N,N*-diallyltosylate (DATA, **1**) in emulsion system, related to Figure 1 and Table 1.

Entry	[Ru] catalyst	loading (mol%)	Time (h)	Yield (%)
1	**Ru1**	1	5	97
2	**Ru1**	0.5	2	41
3	**Ru2**	1	1	39
4	**Ru2**	0.5	2	67
5	**Ru2**	0.1	2	10
6[a]	**Ru2**	0.1	2	1
7	**Ru3**	0.5	5	95
8	**Ru3**	0.5	2	65
9	**Ru3**	0.5	2	75
10	**Ru3**	0.1	2	10
11[a]	**Ru3**	0.1	2	3
12	**Ru4**	0.5	2	28
13	**Ru4**	0.1	2	10
14	**Ru5**	0.5	2	39
15	**Ru5**	1	2	77
16	**Ru6**	1	2	97
17	**Ru6**	0.5	2	77
18	**Ru6**	0.1	2	18
19	**Ru7**	0.5	2	99
20[b]	**Ru7**	0.5	2	98
21	**Ru7**	0.1	2	25
22	**Ru8**	1	5	10
23	**Ru8**	0.5	5	3
24	**Ru9Cl**	0.5	2	32
25	**Ru9Cl**	0.1	2	4
26	**Ru9PF$_6$**	0.5	5	85
27	**Ru9PF$_6$**	0.1	2	21
28	**Ru9BARF**	1	2	99
29	**Ru9BARF**	0.5	2	98
30	**Ru9BARF**	0.1	2	99

Reaction conditions: DATA (0.4 mmol, 101 mg, 1.0 equiv.), durene (0.4 mmol, 54.2 mg, 1.0 equiv.), ruthenium catalyst, and 0.8 mL of non-degassed distilled H_2O was placed in the 4 mL vial and put into ultrasounds' bath at 50 °C for indicated time. Yields were determined by ^1H NMR of crude mixture using durene as an internal standard. [a]Reactions performed in HPLC grade degassed water. [b]Reaction performed on two-times larger scale.

Copies of NMR spectra of new compounds

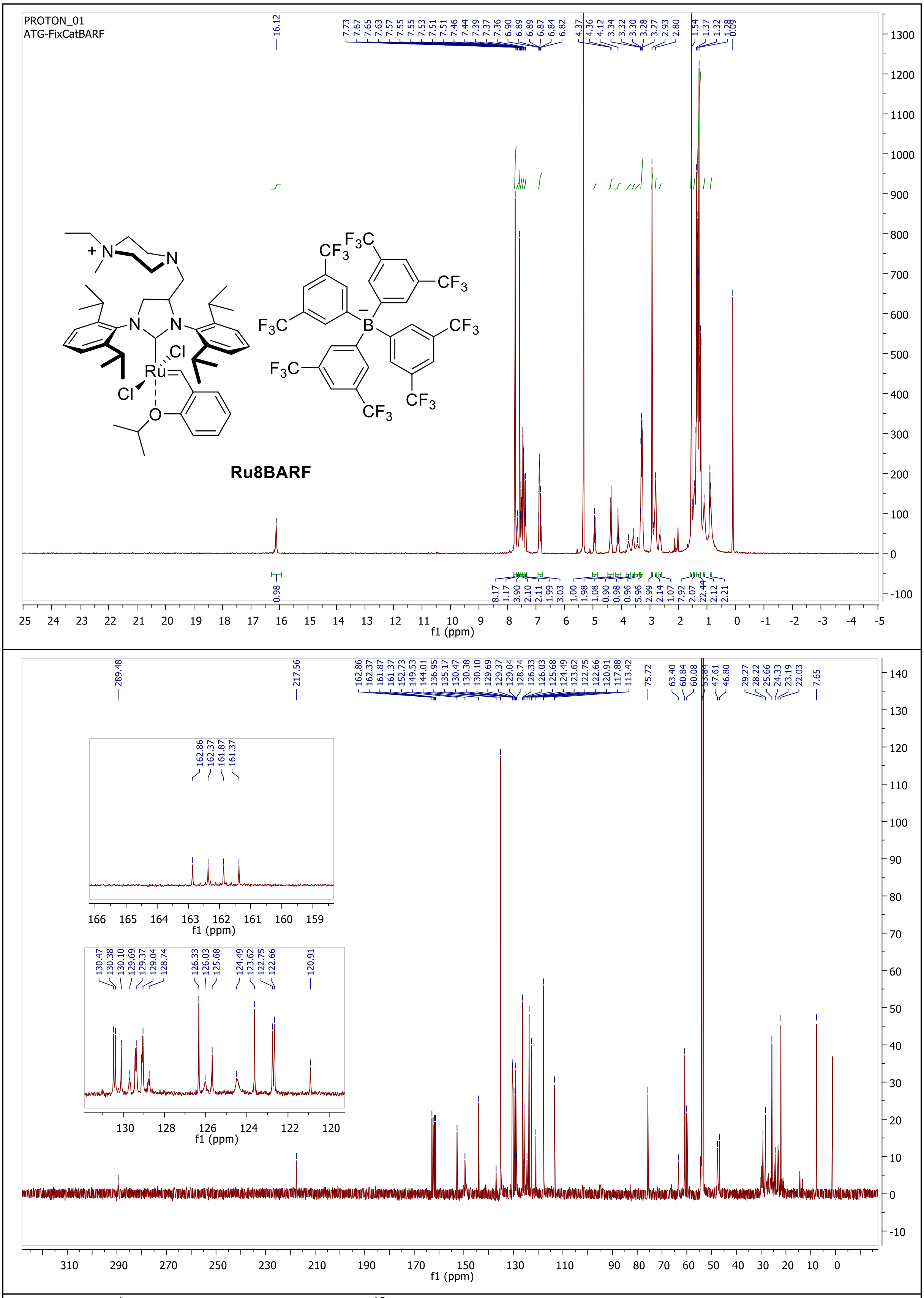

Figure S1. ^{1}H NMR (400 MHz, top) and ^{13}C NMR (101 MHz, bottom) spectra of **Ru9BARF** in CD_2Cl_2, related to STAR Methods.

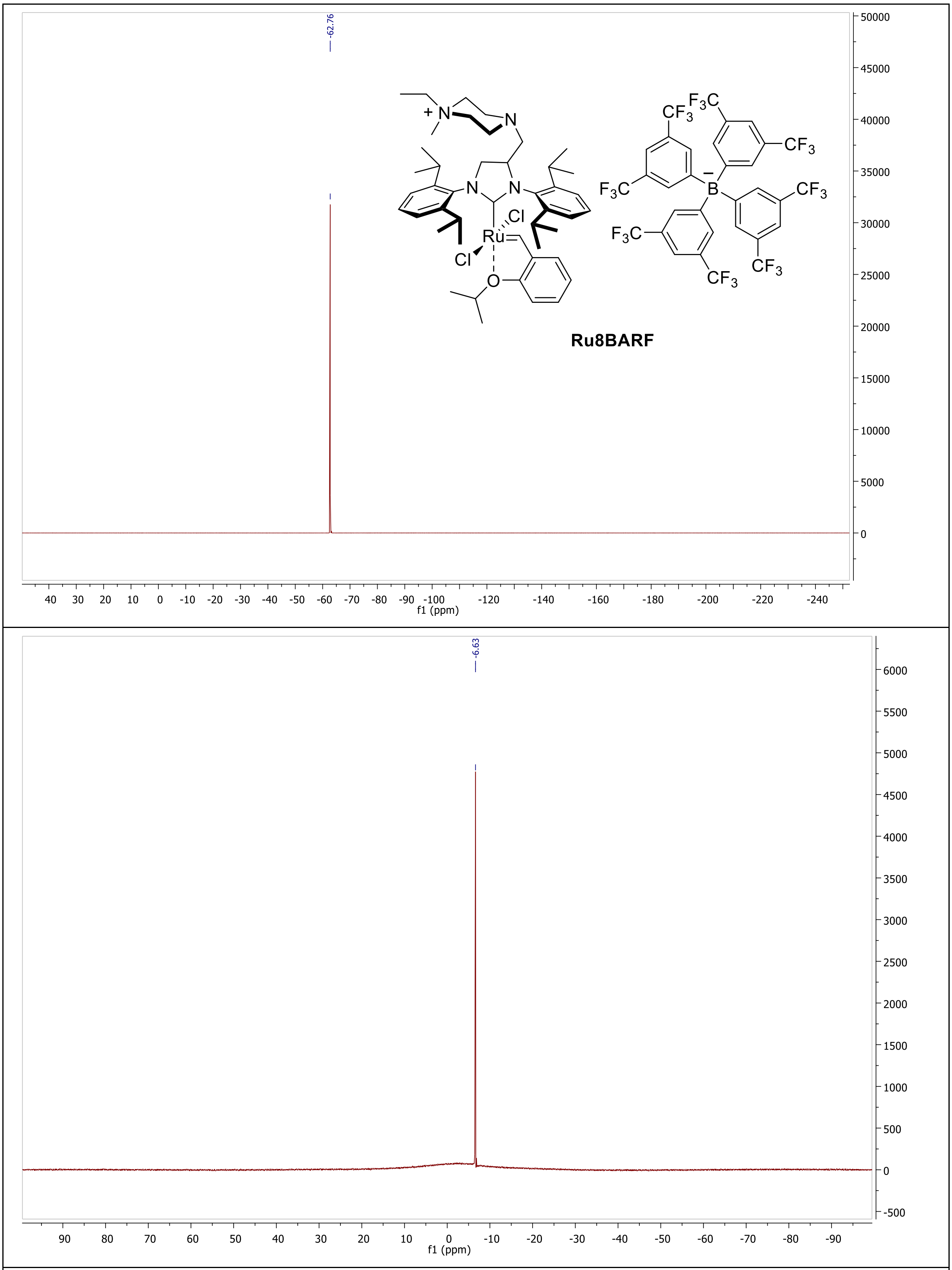

Figure S2. ^{19}F NMR (376 MHz, top) and ^{11}B NMR (128 MHz, bottom) spectra of **Ru9BARF** in CD_2Cl_2, related to STAR Methods.

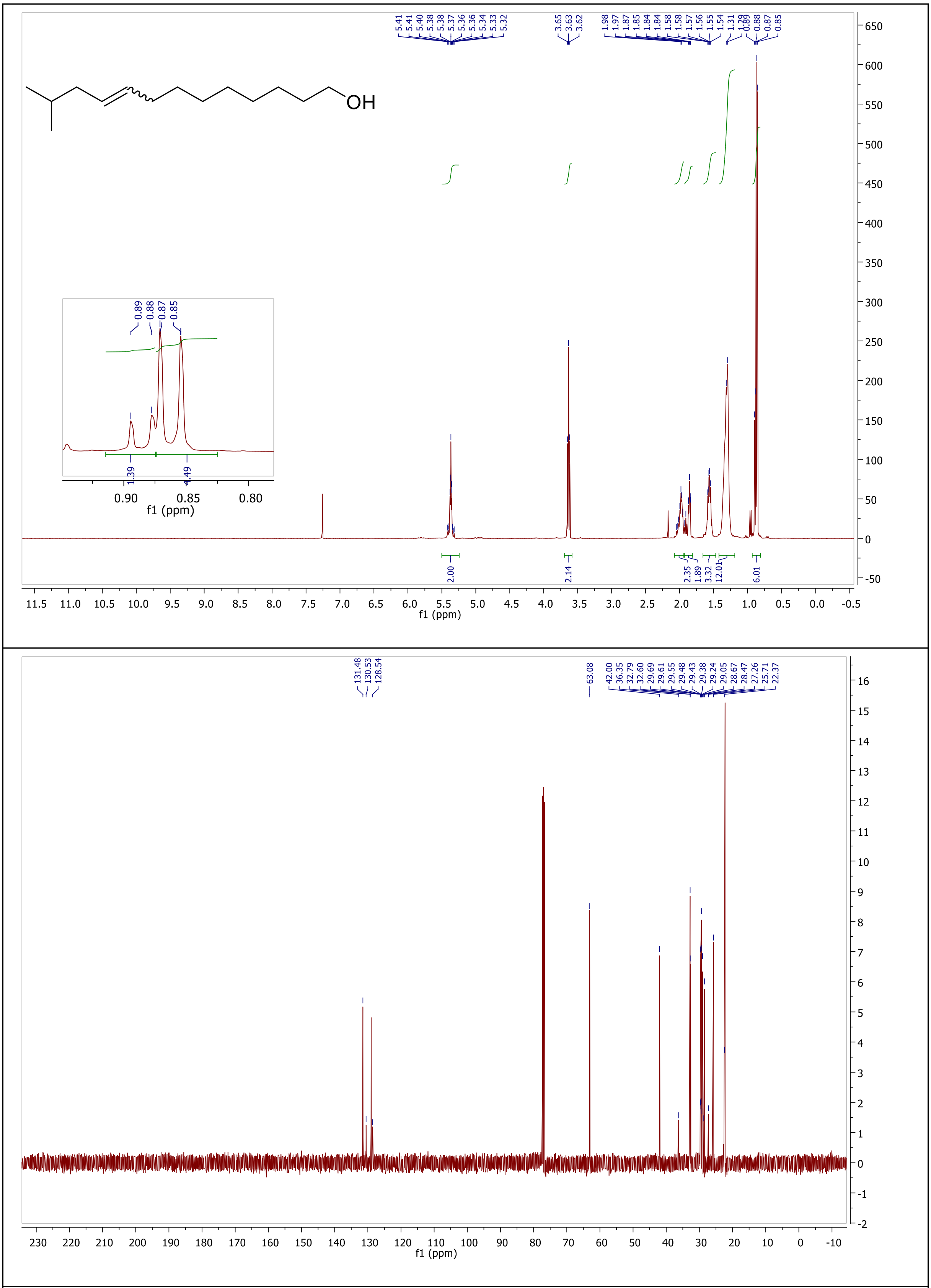

Figure S3. ^{1}H NMR (400 MHz, top) and ^{13}C NMR (101 MHz, bottom) spectra of compound **45** in $CDCl_3$, related to STAR Methods.

Large-scale reactions in the emulsion system

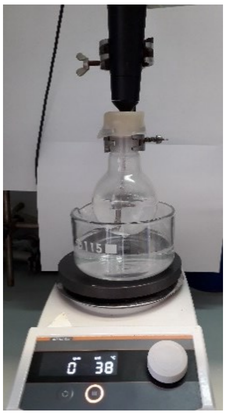

Reaction setup

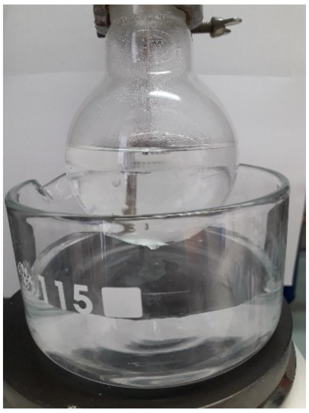

Substrates floating on the surface of water

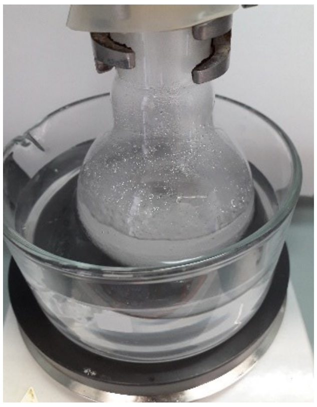

Emulsion formed by stirring substrates with the water

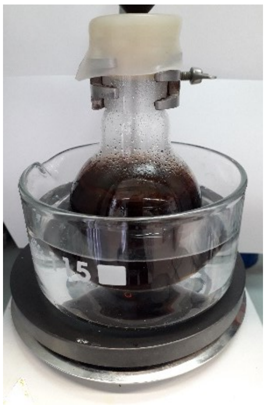

Reaction mixture after addition of the catalyst

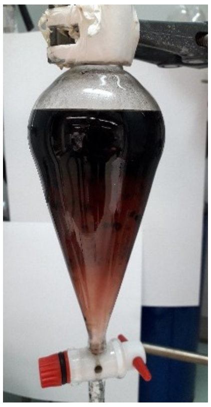

Phase separation

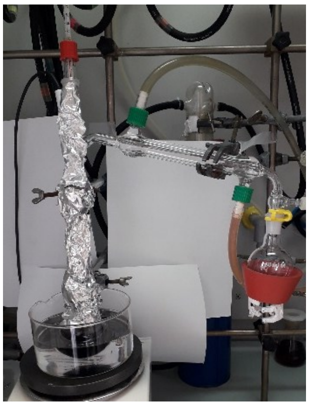

Distillation setup

Figure S4. Large-scale preparation of compound **35** in the emulsion system using a milk frother, related to Scheme 3.

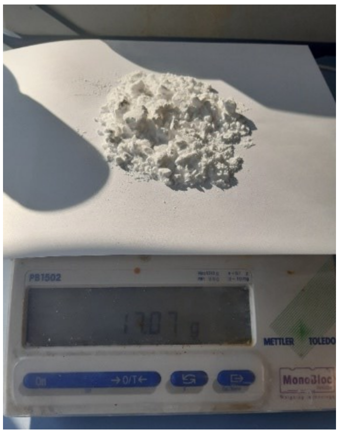

Weighting of the substrate

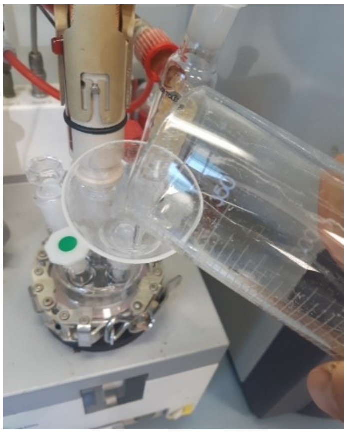

Charging of water

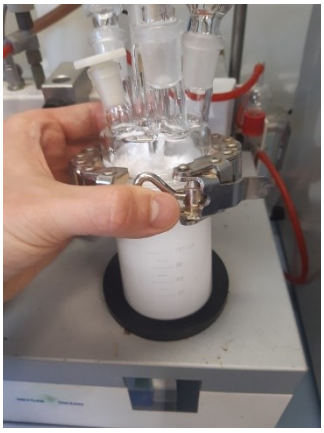

Heating

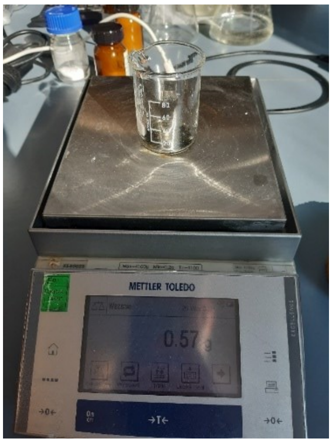

Weighting of the catalyst

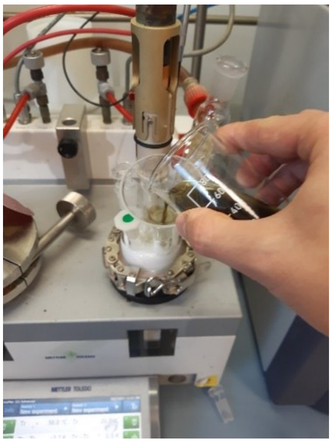

Charging of the catalyst solution

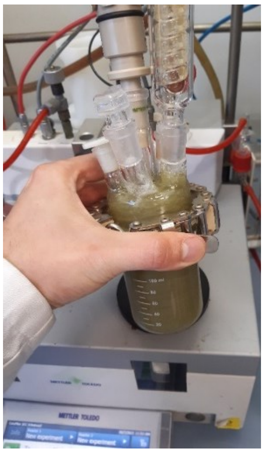

Reaction mixture after 3 hours

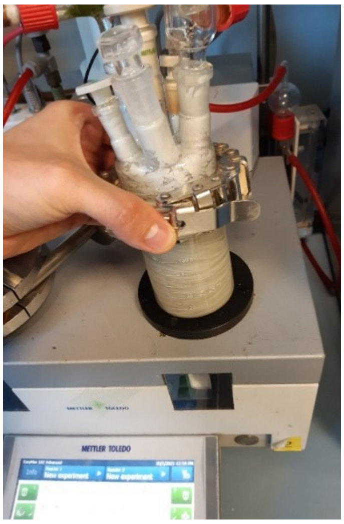

Reaction mixture after cooling down

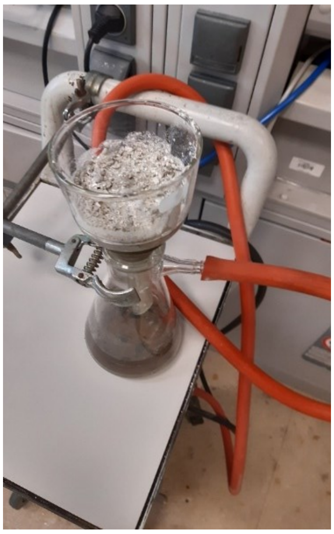

Filtration of the product

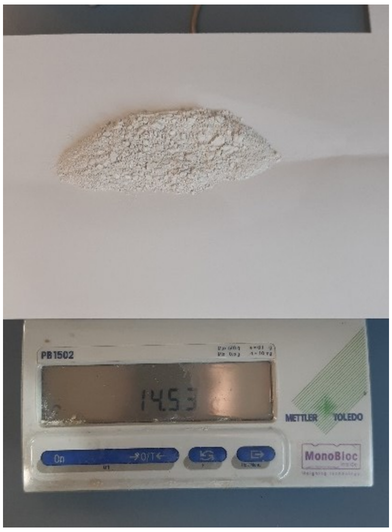

Weighting of the product

Figure S5. Large-scale preparation of compound **24** in the emulsion system using Mettler Toledo Easymax workstation reactor, related to Scheme 4.

Metathesis reaction of tetrasubstituted C–C double bond in the emulsion system

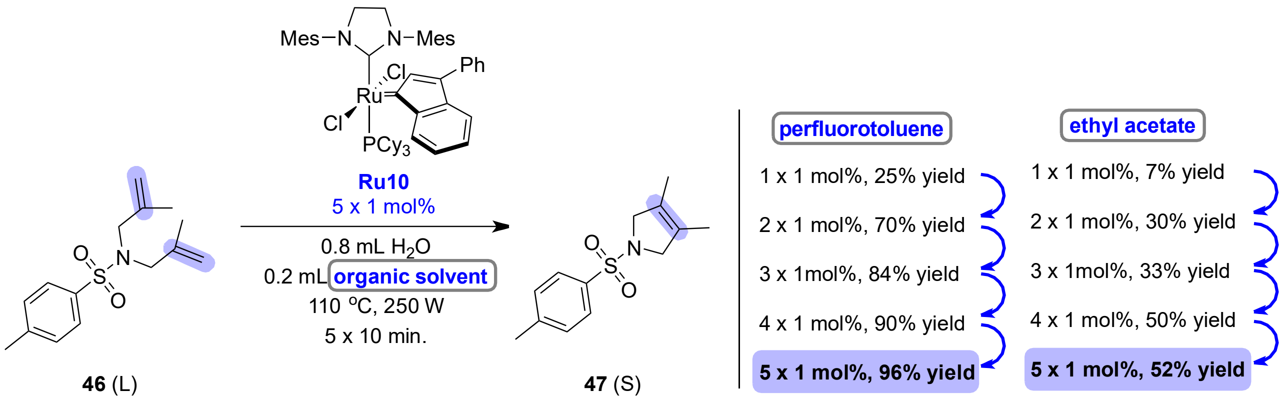

Scheme S1. Preparation of **47** in the emulsion system using microwaves irradiation; reactants are marked as (S) = solid or (L) = liquid, related to Scheme 2.
